# The Escherichia coli Type III Secretion System 2 Has a Global Effect on Cell Surface

**DOI:** 10.1128/mBio.01070-18

**Published:** 2018-07-03

**Authors:** Alexander Shulman, Yael Yair, Dvora Biran, Thomas Sura, Andreas Otto, Uri Gophna, Dörte Becher, Michael Hecker, Eliora Z. Ron

**Affiliations:** a Department of Molecular Microbiology and Biotechnology, Faculty of Life Sciences, Tel Aviv University, Tel Aviv, Israel; b Porter School for the Environment, Tel Aviv University, Tel Aviv, Israel; c Institute for Microbiology, Ernst-Moritz-Arndt University, Greifswald, Germany; GSK Vaccines

**Keywords:** ETT2, Escherichia coli, ExPEC, secretion, T3SS

## Abstract

Many strains of Escherichia coli carry a 29,250-bp ETT2 pathogenicity island (PAI), which includes genes predicted to encode type III secretion system (T3SS) components. Because it is similar to the *Salmonella* pathogenicity island 1 (SPI-1) system, encoding a T3SS in Salmonella enterica, it was assumed that ETT2 also encodes a secretion system injecting effectors into host cells. This assumption was checked in E. coli serotype O2—associated with urinary tract infections and septicemia—which has an intact ETT2 gene cluster, in contrast to most strains in which this cluster carries deletions and mutations. A proteomic search did not reveal any putative secreted effector. Instead, the majority of the secreted proteins were identified as flagellar proteins. A deletion of the ETT2 gene cluster significantly reduced the secretion of flagellar proteins, resulting in reduced motility. There was also a significant reduction in the transcriptional level of flagellar genes, indicating that ETT2 affects the synthesis, rather than secretion, of flagellar proteins. The ETT2 deletion also resulted in additional major changes in secretion of fimbrial proteins and cell surface proteins, resulting in relative resistance to detergents and hydrophobic antibiotics (novobiocin), secretion of large amounts of outer membrane vesicles (OMVs), and altered multicellular behavior. Most important, the ETT2 deletion mutants were sensitive to serum. These major changes indicate that the ETT2 gene cluster has a global effect on cell surface and physiology, which is especially important for pathogenicity, as it contributes to the ability of the bacteria to survive serum and cause sepsis.

## IMPORTANCE

Drug-resistant extraintestinal pathogenic E. coli (ExPEC) strains are major pathogens, especially in hospital- and community-acquired infections. They are the major cause of urinary tract infections and are often involved in septicemia with high mortality. ExPEC strains are characterized by broad-spectrum antibiotic resistance, and development of a vaccine is not trivial because the ExPEC strains include a large number of serotypes. It is therefore important to understand the virulence factors that are involved in pathogenicity of ExPEC and identify new targets for development of antibacterial drugs or vaccines. Such a target could be ETT2, a unique type III secretion system present (complete or in parts) in many ExPEC strains. Here, we show that this system has a major effect on the bacterial surface—it affects sensitivity to drugs, motility, and secretion of extracellular proteins and outer membrane vesicles. Most importantly, this system is important for serum resistance, a prerequisite for septicemia.

## INTRODUCTION

The type III secretion system (T3SS) is an important virulence factor of Gram-negative bacteria that delivers effector proteins into host cells to subvert host cellular processes. In enteropathogenic Escherichia coli, such as strain O157, there is a well-defined T3SS, which is an essential virulence factor involved in attachment and effacement. In many E. coli genomes, there is a similar gene cluster that resembles the *Salmonella* pathogenicity island 1 (SPI-1) system in Salmonella enterica, and potentially encodes an additional T3SS. This gene cluster is ETT2—E. coli type III secretion system 2 (1, 2). ETT2 is present in many E. coli strains, but it usually carries a large number of mutations, deletions, and insertions. So far only a few sets of complete ETT2 have been reported (3–8). The importance of ETT2 in pathogenicity was shown in the E. coli O7:K1 strain involved in neonatal meningitis (NMEC), where it is important in an endothelial cell invasion that simulates breach of the blood-brain barrier during meningitis (9). E. coli serotype O78 carries an ETT2 gene cluster that has a large deletion and several point mutations that result in an inability to produce the needle of the T3SS apparatus. Yet, it was shown that deletion of the whole gene cluster results in reduced mortality of chicks and in increased serum sensitivity (8, 10). It therefore appears that ETT2 is important for bacterial pathogenesis, yet so far its role is not understood, and there is no evidence for the secretion of effectors involved in virulence.

E. coli serotype O2 carries a complete ETT2 pathogenicity island (PAI) (8, 10) and does not have the locus of enterocyte effacement (LEE) gene cluster that also encodes a T3SS (11–15). Thus, ETT2 is the only gene cluster that can potentially encode secretion of pathogenicity-associated proteins. E. coli serotype O2 is frequently associated with urinary tract infections and septicemia and is also often involved in avian colisepticemia. In order to understand the function of ETT2 in E. coli O2, we carried out experiments aimed at identifying the effector proteins secreted by the bacterium and determining the function of this secretion system. We could not identify potential effectors in the secretome. However, we could show that the ETT2 system plays an important role in protein secretion, cell surface composition, and serum survival.

## RESULTS

### The ETT2 gene cluster of E. coli O2.

**FIG 1:**
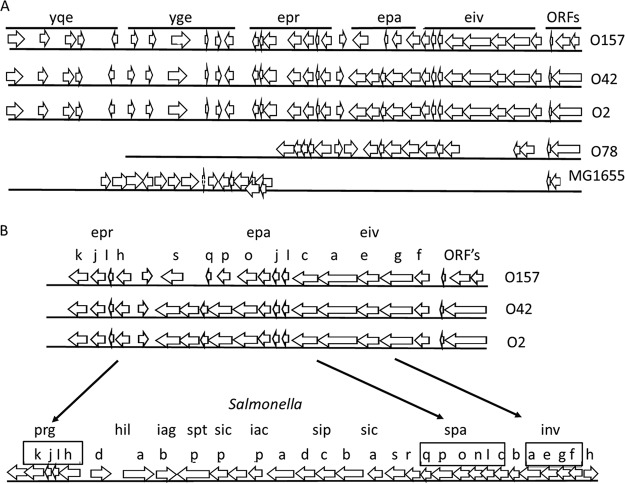
Schematic representation of the ETT2 gene cluster. (A) The gene cluster of several E. coli strains. From top to bottom are shown serotype O157 (1), serotype O42 (6), serotype O2, serotype O78 (8), and K-12 MG1655 (1, 6). On the figure, single lowercase letters represent the corresponding genes shown from left to right from the following operons: *yqeGHIJK*, *ygeFGHIJK*, *eprKJIH*, *epaSQPO*, and *eivJICAEGF*. (B) Comparison between ETT2 and the *Salmonella* SPI-1 cluster.

The sequence of the ETT2 gene clusters of E. coli O2 was compared with previously published sequences from other E. coli strains. The results shown in Fig. 1A indicate that in the majority of the strains, the gene cluster carries deletions. It should be noted that the gene cluster of O2 is highly similar to the complete gene cluster of E. coli O42 (5, 6), and both appear to be complete. The genes present in the ETT2 cluster show high homology to the SPI-1 gene cluster of *Salmonella* in terms of both sequence and order (Fig. 1B) (2, 16, 17). ETT2 contains the *eprI*, *eprJ*, and *eivJ* genes (homologues of *prgI*, *prgJ*, and *invJ*, respectively), which are assumed to be involved in formation of the needle structure, the *eivC* gene encoding the putative ATPase required for energizing transport (a homologue of *invC*), and *epaS* (a homologue of *spaS*), a putative component of the secretion apparatus. Therefore, it was assumed that the ETT2 is involved in secretion of effectors into the host cell, similar to the *Salmonella* system. However, in contrast to *Salmonella*, the ETT2 gene cluster does not contain genes coding for effectors, such as the Sip and Sop proteins, which are translocated into the host cell, and homologues of these genes could not be found in other locations of the E. coli O2 chromosome. Clearly, the possibility exists that effectors secreted by the ETT2 gene cluster exist, but do not have a high sequence similarity to secreted effectors of *Salmonella*. Therefore, we took a proteomic approach to search for potential effectors among the secreted proteins.

### Proteomic analysis of the secretome.

**FIG 2:**
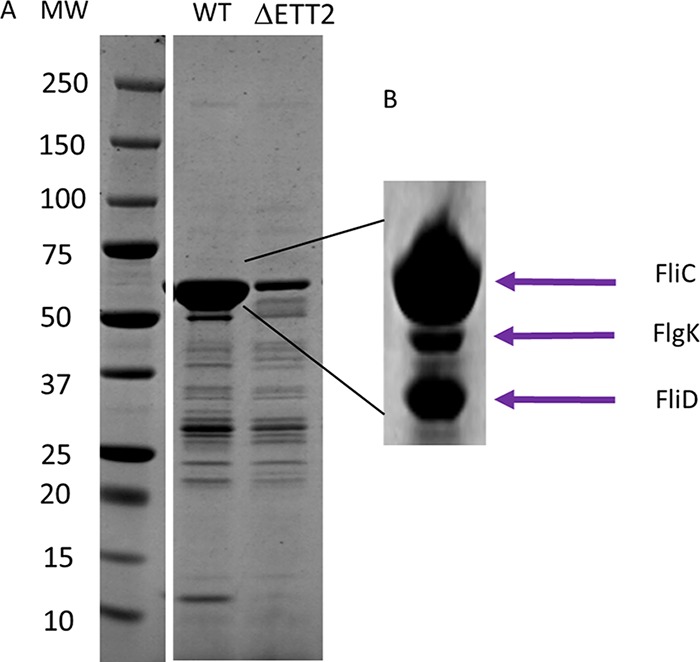
Secreted proteins of wild-type (WT) strain O2 and its ΔETT2 deletion mutant. (A) Total secreted proteins. (B) Identified proteins, MW around 55 kDa. Secreted proteins were prepared and analyzed as described in Materials and Methods. MW, molecular weight.

**FIG 3:**
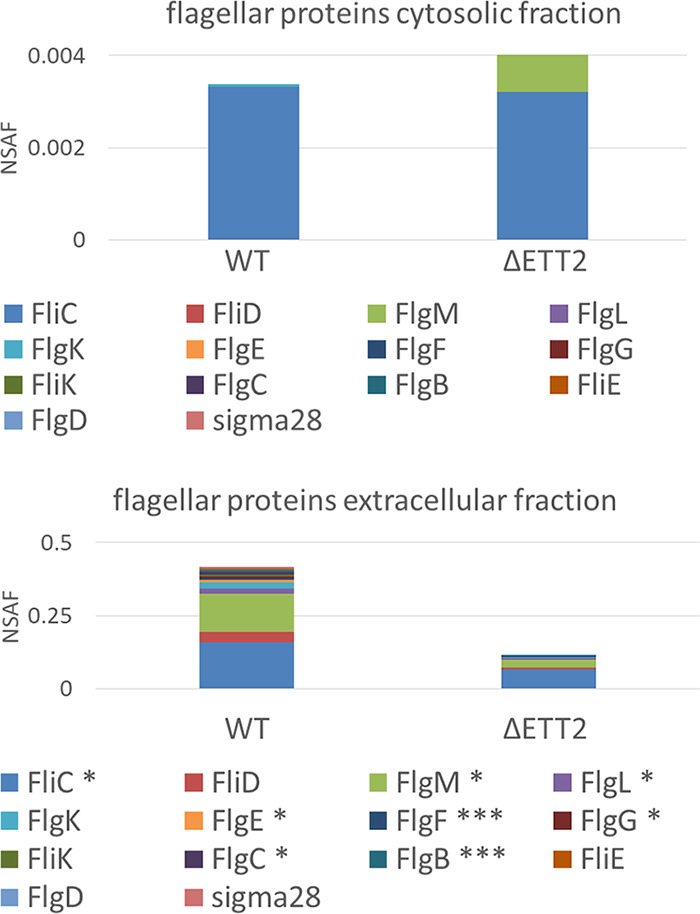
Distribution of flagellar proteins between the cytosol and the secretome of the wild type (WT) and the ΔETT2 mutant strains. Proteomic quantity calculation was carried out using the normalized spectral abundance factor (NSAF) (41). The asterisks depict statistical significance of WT versus ΔETT2: *, *P* = 0.05; ***, *P* = 0.01.

In order to identify putative secreted effectors, we analyzed the secretome of E. coli O2. The secretome contained a large number of proteins that were analyzed and characterized. Basically, they included proteins of the flagellar apparatus, fimbriae, and other known secreted proteins (Fig. 2; see Table S1 in the supplemental material). However, we could not identify a potential effector among the secreted proteins. Therefore, we constructed a mutant with deletion for the entire ETT2 gene cluster and looked for secreted proteins that were absent in the secretome of the mutant. Unexpectedly, the deletion mutant had a much lower level of many secreted proteins (Fig. 2A), including proteins that belong to the flagellar apparatus. These included flagellin (FliC), an assembly protein (FliD), a structural protein (FlgK), and FlgM, the anti-σ^28^ protein that is secreted through the flagellar T3SS, enabling the expression of the late class III flagellar genes. The distribution of flagellar proteins between the cytosol and the secretome of the wild type and the mutant strains is shown in Fig. 3. The results indicate that in the cytosolic fraction of the wild type, only FlgC can be observed, while in the deletion mutant there is also a substantial amount of FlgM. The extracellular fraction of the wild type and the mutant appears to contain all the known flagellar proteins, but their concentration in the deletion mutant is significantly reduced.

10.1128/mBio.01070-18.3 TABLE S1  List of proteins identified in the secretome. Proteins identified with ≥2 peptides. Quantitative values represent the normalized spectral abundance factor (NSAF) as calculated in the Scaffold software suite. Download TABLE S1, XLSX file, 0.2 MB. Copyright © 2018 Shulman et al. 2018 Shulman et al. This content is distributed under the terms of the Creative Commons Attribution 4.0 International license.

**FIG 4:**
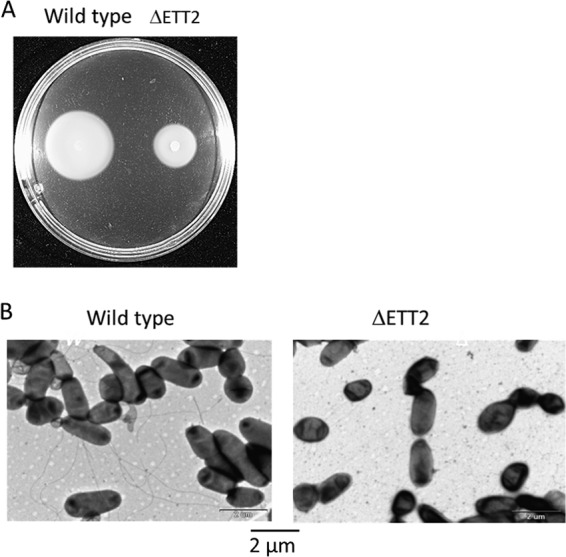
Effect of ΔETT2 on motility and flagella. Motility was measured on minimal 0.3% agar plates incubated for 12 h at 37°C (A). Flagella were observed by electron microscopy (B).

The reduction in secreted flagellar proteins suggested that the ΔETT2 gene cluster affects motility. Indeed, when motility was measured by swarming on soft minimal agar plates, the ΔETT2 deletion was associated with greatly reduced motility (Fig. 4A). The reduction in flagella was determined by electron microscopy. No flagella could be seen, indicating that even if present, they are too few to detect microscopically (Fig. 4B).

The reduced level of flagellar proteins in ETT2 deletion mutants was unexpected. Although export of flagellar proteins is also carried out by a type III secretion mechanism, it uses specific genes, present in the *fli* operon, which are independent of the ETT2 secretion system (18, 19).

### A deletion of ETT2 results in reduced expression of flagellar genes.

**FIG 5:**
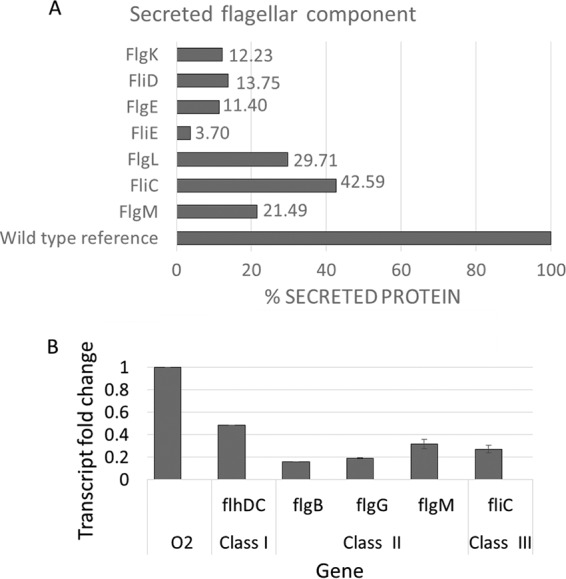
Effect of ΔETT2 on secretion of flagellar proteins and expression of flagellar genes. The level of proteins secreted by the deletion mutant was calculated from the proteomic experiments (A) and is presented as a percentage of the level of each protein in the secretome of wild-type E. coli O2. Expression of flagellar genes was determined by RT-PCR and is presented in relation to the expression in wild-type E. coli strain O2 (B).

**FIG 6:**
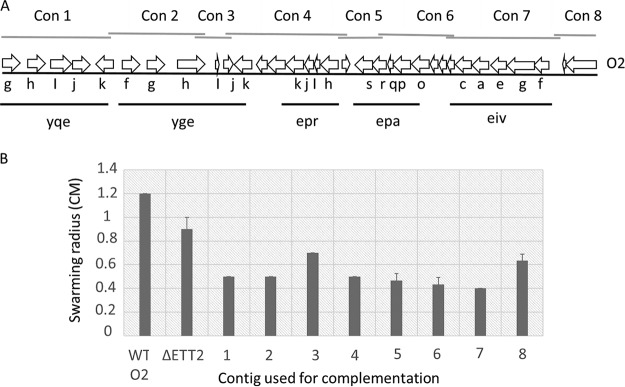
Complementation of motility loss by overlapping contigs that cover the entire ETT2 PAI. Panel A shows a schematic presentation of the eight contigs. Quantification of motility loss was determined by the swarming assay and is presented in panel B. On the figure, single lowercase letters represent the corresponding genes of the operons shown, as described in the legend to Fig. 1.

The deletion of the ETT2 system affects not only the secretion of flagellar proteins, but also their synthesis (Fig. 5). Figure 5A shows that secretion of several flagellar proteins is reduced in the ΔETT2 strain compared with wild-type E. coli O2. Figure 5B shows that the transcription of genes encoding flagellar components is also significantly reduced in the mutant relative to its wild-type parent; this includes the flagellar class I master regulator *flhDC* and representative genes from class II (*flgM*, *flgB*, and *flgG*) and class III (*fliC*). The reduced transcription could be due to loss of activity of a putative flagellar regulator present in the ETT2 gene cluster. To examine this possibility, we constructed eight overlapping fragments that cover the entire ETT2 PAI, as shown in Fig. 6A. These fragments contained all the genes of the cluster, and all of them were intact, as shown in Fig. S1 in the supplemental material. We examined the ability of the overlapping ETT2 fragments to restore swarming, flagellar gene expression or flagellar protein secretion. Our results show that none of the ETT2 fragments could complement the effect of the deletion on swarming or any of the flagellum-related functions. The results of swarming determinations are presented in Fig. 6B. Therefore, we can exclude the possibility that a single gene from the ETT2 cluster regulates expression of flagellar genes. Clearly, more than one gene is required for the complementation, and this gene could potentially be part of the ETT2 gene cluster or could be located outside the cluster and affects the system in *trans*. The regulatory circuits of communication between the ETT2 gene cluster and the flagellum-encoding genes possibly involve more than one gene, at least one of which is located in the ETT2 cluster.

10.1128/mBio.01070-18.1 FIG S1  List of ETT2 contigs. Download FIG S1, DOC file, 0.1 MB. Copyright © 2018 Shulman et al. 2018 Shulman et al. This content is distributed under the terms of the Creative Commons Attribution 4.0 International license.

### Secretion of fimbrial proteins by ΔETT2.

**FIG 7:**
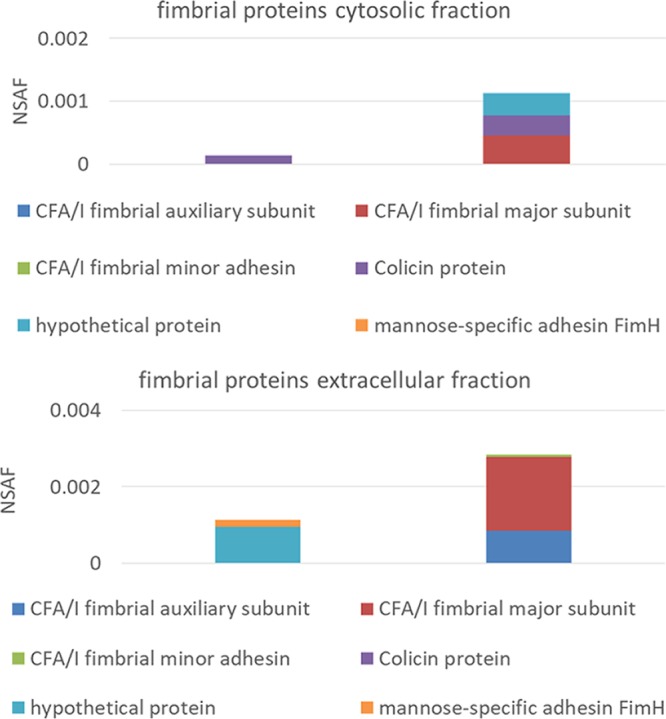
Distribution of fimbrial proteins in the cytosol and secretome of E. coli O2 and its ETT2 deletion mutant. The data were calculated from the proteomic experiments.

The ETT2 deletion affected a large number of proteins in terms of their distribution between the cytosol and the secretome. A summary of the effect of the deletion on the distribution of proteins is presented in Table S1. One of the striking differences between the mutant and the wild type is summarized in Fig. 7, which demonstrates the distribution of fimbria-related proteins in the wild type and in the mutant. Apparently, the mutant accumulates several fimbrial proteins, and the pattern of secreted fimbrial proteins is clearly different from that of the wild type.

### Deletion of the ETT2 gene cluster changes the surface properties.

**FIG 8:**
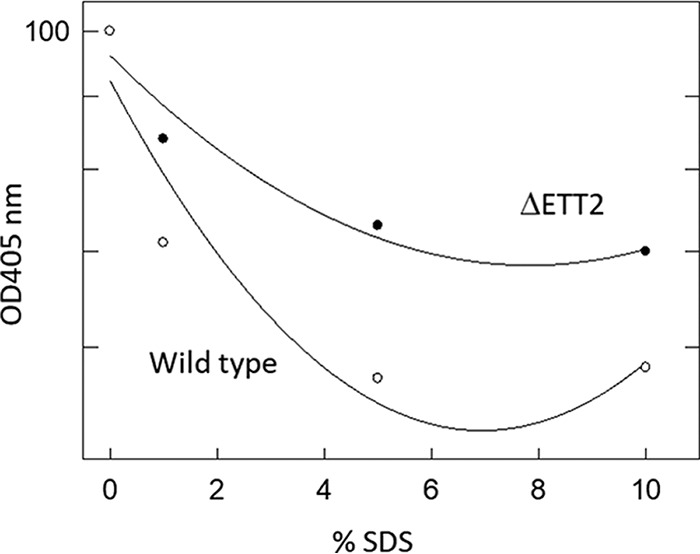
Effect of SDS on E. coli O2 and its ETT2 deletion mutant. Bacteria were grown overnight with shaking at 30°C in defined minimal Davis and Mingioli medium (42) supplemented with 0.005% of each amino acid and with 0.4% glycerol as a carbon source. The cultures were diluted 1:25 into fresh growth medium, grown until they reached an OD_600_ of 0.4, and diluted 1:10 into a sterile 96-well plate for a final concentration of 1 to 10% SDS. The plates were then incubated for 4 min at room temperature, and cell degradation was measured at OD_405_ using a BioTek Eon plate reader.

**FIG 9:**
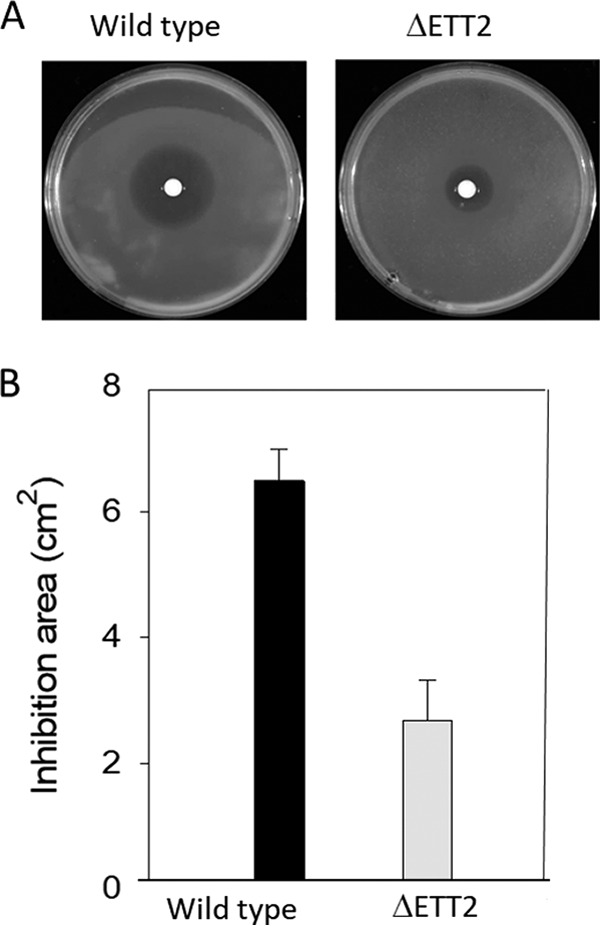
Effect of novobiocin on E. coli O2 and its ETT2 deletion mutant. Overnight-grown cultures were diluted 1:25 into fresh growth medium, grown until they reached OD_600_ of 0.4. One hundred microliters of the cell culture was added into 3 ml of Davis soft agar (0.7%), stirred, and poured on top of Davis plates. Disks 6 mm in diameter (Schleicher & Schuell) were placed at the plate center, and 5 µl of novobiocin (Sigma-Aldrich) stock solution (100 mg/ml) was added to each disk. The plates were incubated overnight at 30°C, and the novobiocin inhibition area was measured. The area of inhibition is shown in the photos in panel A and quantified in panel B.

**FIG 10:**
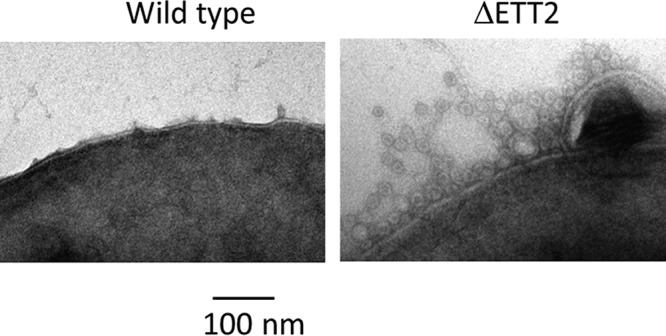
Production of outer membrane vesicles (OMVs). Cultures were grown as described in Materials and Methods, and OMVs were observed by electron microscopy.

As ETT2 is an outer surface-associated system, and deletion of the gene cluster affects the secretion of many proteins, we examined the possible effect of its deletion on the surface properties of the cells. Indeed, cells carrying the deletion of ETT2 have increased resistance to detergents—as seen in Fig. 8, which demonstrates the effect of SDS. In addition, the mutant showed increased resistance to novobiocin, a highly hydrophobic antibiotic (Fig. 9). The deletion mutants also secrete a large amount of outer membrane vesicles (OMVs), as shown in Fig. 10. Such vesicles could not be observed in the wild type.

### Effect of ETT2 on multicellular behavior.

**FIG 11:**
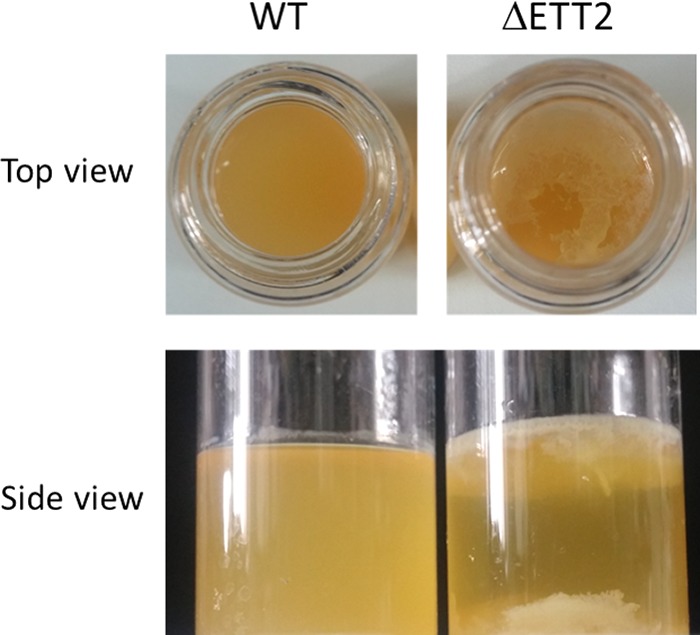
Multicellular behavior in vitro. Cultures were grown in SOBG medium at 30°C as previously described (8, 20).

The results presented so far indicate that a deletion of ETT2 is accompanied by several changes in the secreted proteins, flagellum unavailability, drug permeability, and secretion of OMVs. These major changes also affect the multicellular behavior of the bacteria. The multicellular behavior can be shown by the formation of stable pellicles and biofilms in SOBG medium at 36°C (20). The results presented in Fig. 11 indicate that bacteria with the ETT2 deletion form significantly more biofilm and also aggregate at the bottom of the tube. These represent major differences in the multicellular behavior.

### Effect of ETT2 on serum resistance.

**FIG 12:**
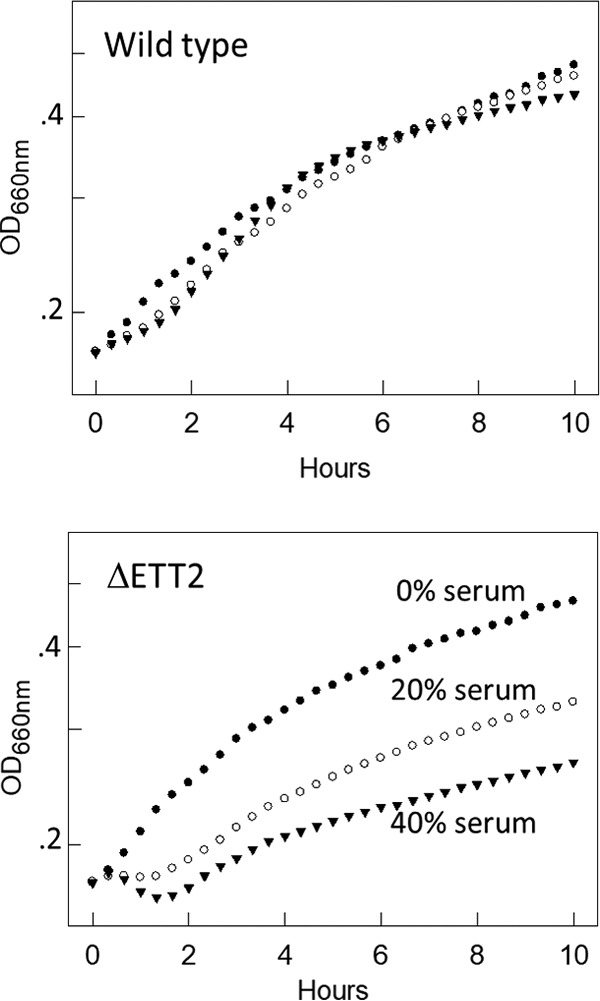
Effect of ΔETT2 deletion on growth and serum sensitivity. Bacteria were grown as described in Fig. 8 and diluted 1:10 into a sterile 96-well plate. Human serum (Sigma) was added to concentrations of 0, 20, and 40%. Growth was determined using a BioTek Eon plate reader, turbidity of 600 nm was measured every 15 min. Solid circles, no serum; open circles, 20% serum; solid triangles, 40% serum.

The deletion of the ETT2 gene cluster had no effect on growth, but resulted in significantly higher sensitivity to serum (Fig. 12). While the wild-type strain was not affected even in the presence of 40% serum, the deletion mutant was already inhibited by 20% serum and even more so by 40% serum. This finding indicates that ETT2 is important for serum survival and is therefore essential for the pathogenesis of the septicemic bacteria.

## DISCUSSION

**FIG 13:**
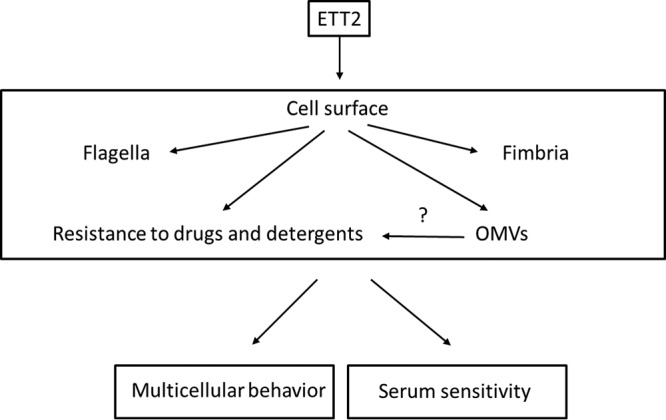
The putative outer surface-related roles of ETT2.

Here we present a study of a pathogenicity island predicted to code for the ETT2 secretion system of E. coli serotype O2. We could not identify potential secreted effectors, as was found for other type III secretion systems. However, we show that ETT2 has a global effect on the cell surface that involves its properties and functions (Fig. 13).

The ETT2 secretion system was first identified in enteropathogenic E. coli O157 (1) and then in a large number of strains. It is highly similar to the SPI-1 system in Salmonella enterica, which was shown to be an active T3SS that secretes specific effectors involved in pathogenicity (21–25). However, the system does not contain genes that are predicted to encode T3SS effectors, and so far no candidates for specific secreted effectors have been identified.

One possible explanation for the inability to identify secreted effectors is the fact that most E. coli strains contain only partial ETT2 gene clusters that have many deletions and mutations (3–8). In contrast, E. coli serotype O2 has a complete ETT2 gene cluster and could therefore be used for identification of specific effectors secreted by this system. For this purpose, we studied the secretome of E. coli serotype O2, grown under a variety of conditions—media, temperature, and aeration. These secretomes were compared with secretomes obtained from mutants with deletion of the ETT2 gene cluster, grown under the same conditions.

The wild-type E. coli O2 strain secretes a large number of proteins, many which are not found in the secretome of the ETT2 deletion mutant (Fig. 2A). These proteins were identified and include proteins with a variety of functions, probably not related to pathogenicity. We could not find any evidence for the presence of secreted T3SS effectors. This result may indicate that ETT2 does not secrete specific effectors. Although we could not detect any secreted effectors or any T3SS components, it is conceivable that we have not yet identified the right conditions for expression and/or secretion of the effectors. Possibly different environmental conditions are required for effectors to be secreted, as described in the case of LEE T3SS (26) or in Yersinia enterocolitica (27).

However, most striking was the finding that the wild type secretes flagellar proteins, which are not secreted by the mutants (Fig. 2B and 3). The reduction of secreted flagellar proteins in the mutant results in lack of visible flagella and reduced motility (Fig. 4). Moreover, there appears to be a regulatory effect of the ETT2 system on the flagellar genes, as the transcription of flagellar genes is greatly reduced in the mutant (Fig. 5). The interactions between the flagellum gene cluster and the gene cluster of the T3SS has been documented previously (for review, see reference 18 by Diepold and Armitage). A positive effect of T3SS on flagella has been reported in *Salmonella* SPI-1, where overexpression of HilD enhances *flhDC* expression, but in contrast to our data, deletion of the entire SPI-1 has no effect on motility (28). In E. coli, the involvement of ETT2 in flagellar expression is probably different than in *Salmonella*, as E. coli does not have the HilD protein or an analogue of it. Also, ETT2 deletion reduces *flhDC* transcription, and its function cannot be restored by complementation with any single fragment of the ETT2 gene cluster (Fig. 6).

Furthermore, recent studies on E. coli serotype O78—which has a truncated ETT2—indicate that the putative ATPase EivC ETT2 gene deletion downregulates the transcription of flagellar genes, and this gene complementation restores the lack of flagella and motility (29). The mechanism in E. coli serotype O2 appears to be different, as the lack of flagella and motility could not be complemented by any one of a series of overlapping contigs that cover the ETT2 gene cluster (Fig. 6).

Previous results indicated that flagella are involved in pathogenicity (reviewed in reference 30). Our finding that ETT2 mutants have reduced expression of flagellar genes and a drastically reduced secretion of flagellar subunits raises the possibility that the reduced virulence and increased serum sensitivity in the ETT2 mutant are a consequence of the reduction in flagellar presence. To examine this possibility, we constructed a mutation in *fliE*, which was previously reported to inhibit the formation of flagellar basal rod and arrests construction of flagella (31), and tested the effect of this mutation on serum sensitivity. The absence of flagella resulted only in partial serum sensitivity, and mutants with deletion of the ETT2 system are much more serum sensitive (see Fig. S2 in the supplemental material). These results indicate that the effect of ETT2 on serum resistance is not only due to the lack of flagella, but is probably due to the additional changes in surface properties.

10.1128/mBio.01070-18.2 FIG S2  Effect of ΔETT2 and deletion of *fliE* on serum sensitivity. Wild-type E. coli O2 and its ETT2 and *fliE* deletion mutants were treated and exposed to 40% serum as described for Fig. 8. Download FIG S2, PDF file, 0.1 MB. Copyright © 2018 Shulman et al. 2018 Shulman et al. This content is distributed under the terms of the Creative Commons Attribution 4.0 International license.

The change in expression pattern and secretion of flagella, although striking, is only one of a series of alterations brought about by the ETT2 deletion. There is a major difference in the distribution of fimbrial subunit proteins between the cytosol and the secretome (Fig. 7). In addition, there are remarkable differences in other cell surface properties—the bacteria are more resistant to detergents and hydrophobic antibiotics such as novobiocin (Fig. 8 and 9, respectively). They also secrete a large amount of outer membrane vesicles (OMVs) (32–35) that are probably also associated with the increase in resistance to detergents and hydrophobic antibiotics. The major consequences of the changes in outer membrane are an altered multicellular behavior and a dramatic increase in sensitivity to serum. E. coli serotype O2 is commonly associated with urinary tract infections and sepsis. The putative roles of ETT2 in balancing the outer surface are summarized in Fig. 13. The loss of serum resistance in ETT2 deletion mutants renders them unable to cause sepsis, as they are cleared from the bloodstream within minutes. Thus, it is clear that ETT2 has a global role in the physiology and pathogenesis of E. coli.

The global effects of ETT2 on bacterial cell surface and serum resistance appear to be independent of secretion of effectors and, therefore, also independent of the presence of the needle structure and secretion apparatus. This concept can explain previous findings indicating that even a deletion of a truncated ETT2 results in loss of virulence and in serum sensitivity (8, 10). It is conceivable that the truncated ETT2, which is not able to code for the protein injection apparatus, can still carry out its role in maintaining the proper structure and composition of the cell surface. The requirement for ETT2, or at least parts of this cluster, for maintaining the physiologically balanced cell surface may explain the prevalence of this system—complete or partial—in E. coli strains.

Our O2 strain contains an ETT2 gene cluster that seems complete, yet no effectors have been identified. These findings raise an interesting possibility—that the system can inject effectors encoded on mobile elements, such as plasmids (as in the *Shigella* T3SS, where both system and effectors are plasmid encoded, or in *Salmonella* where some effectors are prophage encoded), when such elements infect an ETT2-containing E. coli strain. One may speculate that should mobile element-encoded effectors infect the ETT2-containing cell, the system could resume its original role as an effector translocating machine.

Alternatively, in the absence of evidence for secreted proteins, one may assume that this gene cluster—although including genes predicted to code for T3SS—is not involved in protein secretion. Notably, the importance of the genes present in this cluster for motility, cell surface, multicellular behavior, and serum resistance should provide sufficient selection for retention of this gene cluster even in the absence of effector-encoding elements.

## MATERIALS AND METHODS

### Bacteria, growth conditions, and media.

Here we used E. coli serotype O2, which was isolated from a case of urinary tract infection. This strain is virulent to chicks and closely related to E. coli O2 isolates from avian colisepticemia. Unless stated otherwise, the bacteria were grown with shaking at 30°C in defined minimal Davis and Mingioli medium, supplemented with 0.005% of each amino acid and with 0.4% glycerol as a carbon source. Antibiotics were added, when required, at the following concentrations: ampicillin, 100 µg/ml; tetracycline, 12.5 µg/ml; chloramphenicol, 34 µg/ml. *In vitro* multicellular behavior studies were carried out as previously described (20).

### Construction of deletion strains and complementation plasmids.

E. coli serotype O2 was used in this study. Deletions of *fliE* and the ETT2 gene cluster were obtained by the method of Datsenko and Wanner (36). Briefly, competent wild-type E. coli O2 cells were transformed with plasmid pKD119. The transformants were grown in LB medium with tetracycline, induced with arabinose, and made competent for electroporation. A linear PCR product was constructed on the pKD3 template of a chloramphenicol resistance cassette flanked by FLP recognition target (FRT) sequences from the designated deletion region (primers listed in Table 1). Chloramphenicol-resistant cells were picked and examined by colony PCR. The pKD119 plasmid was removed by growth on LB plates at 42°C overnight. The final deletion was verified by sequencing. Recombinant plasmids for complementation were constructed by cloning PCR-amplified DNA fragments (primers listed in Table 1) into a pACYC vector using the Hi-Fi DNA assembly cloning kit (NEBuilder). The cloning products were transformed into competent ΔETT2 02 cells, and the resulting clones were screened by colony PCR. The final construct was verified by sequencing.

**TABLE 1:** Primers used in this study

Primer	Sequence
pACYC F	GCTTCTGTTTCTATCAGCTG
pACYC R	TGGAGATGGCGGACGCGA
#1f	AGAACATATCCATCGCGTCCGCCATCTCCATGTGCGTATCGGTAATGTTC
#1r	ACAGGAGGGACAGCTGATAGAAACAGAAGCTAGAGCAAAATCCGAATTCG
#2f	AGAACATATCCATCGCGTCCGCCATCTCCACTCAATGTCCATTATATACC
#2r	ACAGGAGGGACAGCTGATAGAAACAGAAGCTTGATCAAATTTGGTTCGAT
#3f	AGAACATATCCATCGCGTCCGCCATCTCCACGAGTTATAATTGACAACAT
#3r	ACAGGAGGGACAGCTGATAGAAACAGAAGCCAGAGGACTTATAGATCACT
#4f	AGAACATATCCATCGCGTCCGCCATCTCCACCATATATTCATCCTGAGTG
#4r	ACAGGAGGGACAGCTGATAGAAACAGAAGCAAGAATCTACACGCAAAAAG
#5f	AGAACATATCCATCGCGTCCGCCATCTCCAATCATTACTGGCATTAACAA
#5r	ACAGGAGGGACAGCTGATAGAAACAGAAGCGCATTCGCGAGAGGATAGCA
#6f	AGAACATATCCATCGCGTCCGCCATCTCCAATCCTGGAGTTTCTTTTGTG
#6r	ACAGGAGGGACAGCTGATAGAAACAGAAGCAAAAGAGCTTGAACGAATTG
#7f	AGAACATATCCATCGCGTCCGCCATCTCCACTAACAGTGACTGTAGGTTT
#7r	ACAGGAGGGACAGCTGATAGAAACAGAAGCCATGATGAGGGAAATAAATA
#8f	AGAACATATCCATCGCGTCCGCCATCTCCAGTCCTTGAAGCAAAACATTC
#8r	ACAGGAGGGACAGCTGATAGAAACAGAAGCAATTTATCTTCACCTCATCA
P1TSS2	CATTTGTTATAACTCCGCTCTATCACTTCTCTCGTCGTGTAGGCTGGAGCTGCTTC
P2TSS2	GATTTTCTATATTATCTTAATTCAATCGCTTCAGACCATATGAATATCCTCCTTAG
P1FliE	ATGTCAGCGATACAGGGGATTGAAGGGGTTATCAGCGTGTAGGCTGGAGCTGCTTC
P2FliE	CTGATACGCCGCCACCAGCTTATTACGCACCTGAATCATATGAATATCCTCCTTAG
RTflhDf	TCCGCAATGTTTCGTCT
RTflhDr	ACGAAAGTGACAAACCAG
RTflgBf	GCAGCAAACATCGCCAAT
RTflgBR	ATGTGTTGCGTTGAGGTC
RTflgGf	TATCAAACCATTCGCCAG
RTflgGr	TAAGCGTTCAGTGGCGAG
RTflgMf	CCTCTGAAGCCCGTAAGC
RTflgMr	TGGTGGAGGCGGTTGTTT
RTfliCf	CGTCTGTCTTCTGGCTTG
RTfliCr	AATACCGTCGTTGGCGTT
RTcrpf	GGCAAACCGCAAACAGAC
RTcrpr	GCCCAGTTCGCCAATAAA

### Conditions for growth and determination of serum and SDS survival.

Overnight-grown cultures were diluted 1:25 into fresh growth medium, grown until they reached an optical density at 600 nm (OD_600_) of 0.4, and diluted 1:10 into a sterile 96-well plate. Growth was determined using a BioTek Eon plate reader, and turbidity at 600 nm was measured every 15 min. Serum survival was determined by turbidity measurements following addition of serum. Resistance to SDS was determined by adding SDS (Sigma-Aldrich), incubating the mixture for 4 min at room temperature, and measuring cell degradation by reduced OD_405_ values (37).

### Novobiocin resistance.

Overnight-grown cultures were diluted 1:25 into fresh growth medium and grown until they reached an OD_600_ of 0.4. A 100-µl aliquot of the cell culture was added to 3 ml of Davis soft agar (0.7%), stirred, and poured on top of plates containing Davis agar. Disks 6 mm in diameter (Schleicher & Schuell) were placed at the center of the plate, and 5 µl of novobiocin (Sigma-Aldrich) stock solution (100 mg/ml) was added to each disk. The plates were incubated at 30°C overnight, and the inhibition zone of novobiocin was measured.

### Secretome collection by StrataClean resin beads and SDS-PAGE.

Extracellular proteins were purified using StrataClean resins as previously published (38). Briefly, 50 ml of the culture at OD_600_ of 0.4 was centrifuged at 8,000 rpm at 4°C for 10 min, and the supernatants were ultrafiltrated using 0.45-µm-pore filter, added to 20 µl of StrataClean resin beads, and incubated in a rotation wheel at 4°C overnight. The samples were then centrifuged at 8,000 rpm for 45 min, and the beads were collected into Eppendorf tubes, centrifuged once more at 8,000 rpm for 5 min, resuspended with 10 µl of sample buffer, and incubated at 95°C for 5 min. The samples were then sonicated for 5 min. Thirteen microliters of resin beads was loaded onto 7 to 20% GeBa gel (Gene Bio-application) and separated by electrophoresis.

### Proteomic analyses.

Samples were prepared for liquid chromatography-mass spectrometry (LC-MS) analysis as described elsewhere (39). For secretome analysis, the 7 to 20% GeBa gels (Gene Bio-application) were stained for 1 h with Coomassie brilliant blue G250, distained with acetic acid-methanol solution, and cut into equal-size pieces. Each piece was transferred to a labeled Eppendorf tube and washed three times for 15 min with 700 µl of gel washing buffer (ammonium bicarbonate-acetonitrile) at 37°C, with occasional shaking. The samples were further desiccated in a vacuum centrifuge at 30°C, supplemented with 0.002% of modified trypsin (Promega, Madison, WI), and incubated overnight at 37°C, and the excess trypsin was removed. The pellets were resuspended with sterile, double deionized water and ultrasonicated for 15 min. The samples were concentrated to a final volume of 10 µl by a vacuum centrifuge. The LC-tandem MS (MS/MS) measurement procedure was performed as described elsewhere (40). Further data analyses were conducted using Scaffold (version Scaffold_4.4.6).

### Motility assay.

An overnight culture was diluted 1:25, grown to an OD_600_ of 0.4, and then diluted 1:100 and plated on a 0.3% minimal agar plates supplemented with glycerol and amino acids. The plates were incubated for 12 h at 37°C, and the radius of the colonies was measured.

### Electron microscopy.

Cultures were grown as under the growth conditions described above, and samples were placed on 200 mesh Formvar-coated copper microscopy grid, and after a brief incubation at room temperature, the samples were stained with 2% aqueous uranyl acetate for 30 s. After drying, the samples were observed in a JEM-100B (JEOL) electron microscope at 80 kV. At least 10 bacteria of each culture were screened each time.

### RNA isolation and purification.

Cultures were grown as described above until they reached an OD_600_ of 0.4. Three milliliters of the cultures was rapidly chilled on ice, centrifuged at 8,000 rpm at 4°C, and washed once with cold sterile saline (0.9% NaCl). Pellets were resuspended in 500 µl of minimal medium mixed with 1 ml of RNAprotect bacterial reagent (Qiagen) at room temperature. RNA purifications were conducted using the RNeasy minikit (Qiagen) according to the manufacturer’s instructions. DNase treatment was performed using RNase-free DNase (Qiagen) according to the manufacturer’s instructions.

### Real-time PCR.

Real-time reverse transcription-PCR (RT-PCR) was performed with 1 µg of total RNA, and reverse transcription was carried out using random hexamers (Amersham) with ImPromII reverse transcriptase (Promega). Quantitative PCR (qPCR) was performed using SYBR green PCR master mix (Applied Biosystems) in a total volume of 20 µl. Reactions were carried out on an ABI 7700 instrument (Applied Biosystems) using the standard cycling parameters. Every sample was examined at least three times in duplicate, and the results were normalized to a housekeeping gene (*crp*).
