# Supplementary material for: The Escherichia coli Type III Secretion System 2 Has a Global Effect on Cell Surface
Source: mBio. 2018 Jul 3;9(4):e01070-18. doi: 10.1128/mBio.01070-18 (PMC6030553; doi:10.1128/mBio.01070-18)
Supplement: FIG S1 [file mbo003183965sf1.doc]

Fig. 1 Appendix

**ETT2 contig library**

**#1 ~3700bp**

tgtgcgtatcggtaatgttctttagctaatatccattgtgaatggttcaaaattgctaca

attttgaaccattccagttttcataatgttcatttgttataactccgctctatcacttct

ctcgtcatatcctagtattatcagttagttaaacacacaaagagatctgaacatcttaaa

taagactatttaagatgcataacttagattcgcaagatatcttctggtcattcaaaaaca

atttgcgatatagatattctcataccatgagatagttatctgaaccaccatcaaccaggt

aagattaaatcagtgaaacataatcatatttaataacgagaaatgcatttttaaatgctt

tactcagaattatagcaaattcagattaatcgcaatttttcccagcaaatttacaaaatc

atggtgtattgacatcatcaataaccaatgaaattatgcaattatatacggatagggagg

ttcttaacatggggctttgtagtcgttataaaagtcttacatgcaatagttgctcaatgc

attgccaaataatgccagaagagtcaccgcgtttgcagtattgcgcaaactcgtgttttt

gtatgtggcccgaagaaagttcatattttaatcgtggtgtggtggaaggtattttaacaa

hypothetical protein

yqeH protein

Helix-turn-helix domains

aaaaccacaatgcgagattaagcggatatatttttgtcgatttttcagtaagttttttac

gtctattccttgaaaaagactggattgactatcttgcaagtactgacatggggattgttt

tagtcagcgaccgcaatatgcaatcgttagctaactactggcgaaaacacaactctgcaa

tatctgctgtcatatataatgatgatggtcttgatgttgccaatgaaaagatcagacaac

tatttattggtcgttatttatcatttaccagagggaacacattaactcagatggaattca

ccataatgggatatatggtttctggttataatccatatcagattgctgaagttctggata

tggatatccgtagcatctacgcgtacaagcaacgaatcgaaaagagaatgggtggtaaaa

taaacgagttatttattcgttcacattcggtccaacattgatactaaataatctgccaga

atcatactctgcttatacgggcatctattgaaatgcccgtattttacaaataagcagtaa

ccccaagagtaaacaacatggtatttaaaaccatcacacgaaaaattcagaagcattacg

aaatatgaatttacaaacaatagtggcataaatgttaatcatgttaatttacgtaaagtt

ttacgttgcgacattaaagcctcatttcaatcattatgataaatataaaattaatatata

tttatgccgtaaataccaatatacttagcaaactatgtgatttccattttgattgattta

gtgtctattgacgtatgtactggattattaacgataatatcgagttctggcctgagcacc

gaaaattaatatcggtacataacgccgatcttaacgtcgttctgacaacgccagccagtc

gttgtttatcacttctacttgaagcttttcctgatgtggttgcacaacaagattttttca

ccagagtctgggaagaagaaggtatgcgtgtgccaactaacacgttatatcagaacatat

ccattatcagacgcggatttcgcgctgttggtgataccacccactcgcttattgcaaccg

putative transcriptional regulator

yqeI protein

tgccgagaagaggattcaagattcataatgacattaacatacaaaatcatgtaataaact

cttcaacagacgcacatacacacaatgccccacctgccataaaaggtaatgcggggtaca

aagagagcattggtggcgcaaacaatttcaataacaaaatcctcaaacatataaaaagtt

atctaattatgttgagcatatttgctataggcgtatattctgcacattggctatggaata

ataatcaaccgaagccattcttcaaggattacaagactgttgcggaaattaatggctgcc

attttaatgtgacagaagatacaattgatgggttgaaagagttcgataaatataagacac

ggatactggattcggggattaactgtaaaaaatatccgtggttatacttccctcttgcaa

aatcctcaccagggatgattgttatggcatgcaataaaaactataaccaacatgaagtgg

caggctgtttaaccctgtcttaccgtgaggttaatcgtgattgattataaaaaaacttat tatttattttagtttttatcagtgggtttattctcttcattgtttactcttatactgcag

hypothetical protein

yqeJ protein

aaaaaatgatatataatgaaacatgcactgcaaattgggtaatttttaatgacaaggggc

gagcaaatcttaccatcgactttatgtataaccaaaaaaataaaacaggaactgttgcgt

tgagcggaacctggcaacaaggtaacagagaaagtaagtcaatacggcgaaatattgaat

acacatggattgaaaactatgacacagcccatttaacatcaaagaaagttaataaatttg

agatcatggatcaagtcgatgatgatagacttgcagaacttattcctgatttttatgtct

ttccagaaaaaagtgtaagctataatatactaaagcaaggtaagcatgcttttattttga

gtattggtaacagagcaataatgcattgtgcaaggtaaacttaaatagataataaaaaga

tagaacctcgttaatcataaaacaataaaattaaatatgcttgtagtatatgccagtata

aagcatcaaagttttgatcaacatctttcattttagatatcaccttgcaatgcaaaatat

catgtatcaacaacatctggtgcaaatccatgagtcgaactcaacattcatcttagctct

attattattccttcgtacagtcgatgtaaacaatacagaaagaggattattaagaaccag

tttacttagagaatcaatagaggaaaggactaacgtttctttaaaagaattgatttcatc

conserved hypothetical protein yqeK protein

atctgttaaactaaactcatcattgacagatcgtgagatataactgtttttaactttact

ctttacgttgactttactgacagaattaacattcacatatcttgaatttagtacccatga

tgttgtttgcgtgagaactttctcagcattaaggaatttttttaacggaatccttggttt

ctttttcgagtccgaatttacaatatcacgcatatagaccatttcatgaatctgcgaaaa

ctcaatgtccattatatacctcacattttgaccgtgactcgatgttactgttcaataatc

accttccatcaatactaaatttaatacccctaatgtgccgataacaaatatagtcattct

acgtaacgtctccataaggtgataaccttgatcaaatgaatttcgagaataataaaggaa

atcttttgagatgtttgtcacgaataaagctatttatgttaccaatatcatttcgagtgt

gcacactatctatctgttttttcaggatataaaaaacaacatgtaagttatgtaaattgc

cgatgccactaagtaacattatggtaaaaaaagtaaaaatgagcgatttttgatagaata

aagcatgagaaaaggtgatatttgacattatcagaagctgcgaattcggattttgctcta

hypothetical protein

yqeF protein

domains

#1F TGTGCGTATCGGTAATGTTCTT

#1R TAGAGCAAAATCCGAATTCG

**#2 ~4000bp**

ctcaatgtccattatatacctcacattttgaccgtgactcgatgttactgttcaataatc

accttccatcaatactaaatttaatacccctaatgtgccgataacaaatatagtcattct

acgtaacgtctccataaggtgataaccttgatcaaatgaatttcgagaataataaaggaa

atcttttgagatgtttgtcacgaataaagctatttatgttaccaatatcatttcgagtgt

gcacactatctatctgttttttcaggatataaaaaacaacatgtaagttatgtaaattgc

cgatgccactaagtaacattatggtaaaaaaagtaaaaatgagcgatttttgatagaata

aagcatgagaaaaggtgatatttgacattatcagaagctgcgaattcggattttgctcta

hypothetical protein

yqeF protein

domains

atcaaatgagagaaatgtaaaatgaagccacgaaatattaataatagcctaccactgcaa

ccattagttcctgatcaggagaacaaaaataagaaaaatgaagagaaatacgtgaatcca

gttaaaattacgatgaggtctggtttaaattatattgaacaagaatctcttggaggaaaa

tatctaacacatgatttgtcaataaagatagcggatatttctgaagaaataattcagcaa

gcaatattatcggctatgagcatatataaatttccgataacagatgatttaatgagtatg

gctacaaatgaactcataaaactggccaaaatagagaataatttagacctgaataaattc

tctactatatgcaccgacgttctatcccccctcgtcaccagacataataaagaaaaaaac

tagacgacttactacccttcgcaaaaatcccctttttaattttcatcgaaaaaagggaaa

ttgataatatgaatcaaggtcatgcatattaaggcatttatgaaatatcgtaaataagat

aactaataaggtgaatattagcaaaacaactaatgacattattggtttatagtggtcagt

atttttactaataatgaaccaatatacccttaagcaatgcaagcttaaaacccataattc

aaaaaagataaatgcaaaaaaataatgagtcaattaataataatttagcatcacgaaaca

catcacagaggaatattatgagcacagatacacttgaaatattcaataacagtgatgaat

gggcaagtcaactaaaacacgcattatcacaaggagaaaatctggcattactacatggat

chaperone protein SicA

Tetratricopeptide repeat

ygeG gene product

type III secretion low calcium response

chaperone LcrH/SycD

tgactcctgatatccttgatagaatatatgcatatgcattcgactaccatgaaaaaggta

atataacggacgcagaaatttattataaatttctgtgcatttatgcgttcgaaaatcatg

aatatctaaaaggttttgcatcagtatgccagtcaaaaaagaaatatcaacaagcatatg

acctttacaaactaagttacaattactccccgtatgatgactattcagttatttatcgta

tgggtcaatgtcaaattggggctaaaaatatcgataacgcaatgcaatgtttctatcata

ttattaacaattgtgaggatgaaagtgttaagagcaaagcgcaggcatatattgaactct

taaccgataattcagaagataatggctaagcattacaagcatttggtaaaaattgctggt

tatagtgcagttaaccactatgaccagcttctcttatgttttcttaaatcaacatcttaa

aacagttaagaacacactataaattactatttttcaacgcgttagagaacaatctatttt

atcaaaattgaataaggctatttcagattacatcactgaattcctaagataaattagtga

acacaaccatagatatgtcgaatgtaaactcctgtagcattaatgacacataaatatgaa

tagccacaatttctattgcttatgcaggatgcaagaaaccaattttttcatagaggttaa

ctaatggacttagaaaataaattctcatatcattttctcgagggattaacgctcacggaa

gatggaattcttactcaaggaaatgagcagatttatattccaccgaaagagttaggagta

ttgatcgtattacttgaatccgctggtcatgttgtcctgaaagatatgatcatcgaatca

gtatggaaaaatattattgtaagtgacgagtccctgacaagatgtatctattctttgcgc

ygeH protein

Effector domain of response regulator. Bacteria and

certain eukaryotes like protozoa and higher plants use

two-component signal transduction systems to detect and

respond to changes in the environment. The system consists

of a sensor histidine kinase and

tgcatttttgaaaaaattggctatgatcgttgcatagaaacaatctaccggaaaggttat

cgtttcagtggacaggttttcaaaactaaaataaatgaagataacacttcagactattcc

atagctatatttcctttcaccacttcattgaatactctggatccattaatacttaatcag

gaattagtgcaaaacatttcgaataaaaaaatcgatggcctttatacctatccgatggct

gcgacaaatttttgtaatgatcacatatcgcaaaattcatttttgagaagattcaagcca

gattatttcgttacaggaagaataaaccagaataacgcggtgaaaactttatacattgaa

ctgatcgacgctaaaaatcttttcctcatcgccagtaatcatcttcctgttgatgaacta

cataacacatcacaatttattatagataatatccttcaaacggtacataatccagagcgt

tctgtaagattagctaaaccggaccaaggttataagaatcattatttatcagatgaactg

ttggctggtaagaaagaactttacgagtttactcctgaaagcatttacagggctatggct

atatttgatggattacaaaataaaagtgatatacagacgctaaaaatagaatgttattgt

ctgctagcggaatgccatatgtctttggcacttcatggaaagtatgaacttgaacttgct

gctcaaaaagcattagagcttttacattatgtatcagacataaccaatgtcgatggaaaa

attttagctattatgggactgataactggtctgtctggacaagcaaaagtatctcatatc

ttatttgaacaggctaagatacacacaactgatatagcctctctctactactatagagca

cttgtcaattttcataatgaaagaattgaagaggcaaggatttgtatagacaaatcacta

caactcgaacccagaagacgaaaagcagttgtgataaaagagtgtgtagatatgtatgtg

cctaacccgctcaaaagcaacatgaaactctactataaagaaacttcgagtgaaagccat

cgagttataattgacaacattttgaaactaaagcaactgacgagaatttgtatgcgataa

atctttattgtcgcaattttattgtttagatgtatgcaactggttatgtcaacaacagag

atgaaaacatctaagataattaccttattgcaatatatatcaaatactctagattaaaaa

gtattgtgtataggctattggcctggacaaataaaaaagggaattttatgactaatccaa

hypothetical protein

ygeI

tcaatatgaataatttttctcaaagtttaaatatagcaaccgcaacgggtgatgaagtcg

tttcattagataaacacatcaccaccagtactactgatacagatcaaatacaagcattta

tagtttcaacatggatggcatcttttcaaaatgatatgtattcgaaagataatcctatct

ctccttactatgaactggaatggtagataattgaccacttaaatgattaacgagatactg

aatatattagtatctcgttcctttccttcaatcctacacataaaaatatatgcgcttacc

caaagttaaaatcttatccatcttcatgttttctatattaattacaaaaatgtctttcgc

cagtagcgcttgctttaatgaagcaggatcaattttcagaatcgaaccaaatttgatcaa

#2F CTCAATGTCCATTATATACC

#2R TTGATCAAATTTGGTTCGAT

**#3 ~2000bp**

cgagttataattgacaacattttgaaactaaagcaactgacgagaatttgtatgcgataa

atctttattgtcgcaattttattgtttagatgtatgcaactggttatgtcaacaacagag

atgaaaacatctaagataattaccttattgcaatatatatcaaatactctagattaaaaa

gtattgtgtataggctattggcctggacaaataaaaaagggaattttatgactaatccaa

hypothetical protein

ygeI

tcaatatgaataatttttctcaaagtttaaatatagcaaccgcaacgggtgatgaagtcg

tttcattagataaacacatcaccaccagtactactgatacagatcaaatacaagcattta

tagtttcaacatggatggcatcttttcaaaatgatatgtattcgaaagataatcctatct

ctccttactatgaactggaatggtagataattgaccacttaaatgattaacgagatactg

aatatattagtatctcgttcctttccttcaatcctacacataaaaatatatgcgcttacc

caaagttaaaatcttatccatcttcatgttttctatattaattacaaaaatgtctttcgc

cagtagcgcttgctttaatgaagcaggatcaattttcagaatcgaaccaaatttgatcaa

agctatcgctcttgttgaaagtaatttaaaaaatgatagcatcggcaaaaacagggataa

putative peptidoglycan-binding-like protein

invasion protein IagB [Salmonella enterica subsp

gaaaaacaatataaagagtgttgattatggtttaatgcagataaaccagatgcacattcc

catgttaaaaaaacgtggaataataaaagatgaacgagatttgttagataacccctgtct

gaacattaaaatagggaccgagattttatataatcatttttcacgttgtggggtaacgtg

gcaatgtctggggacatataacgcgggttttgcaatggaaaacaaaaaaaagaggctgca

atatgcaaaaaaaatatatgttgtatataccagacttaatgaaatagacaagcgtaaggc

tttagcaaaataaacctaaagcctcttttttttatatagtgcaaacacccatacgtaaag

ctgttttgatcaactcgataccactgtgaacatctaatttcttcatgatattgagtctat

gagtttcgactgttttcctgcttaactgcaagaaattagcgatttctttgtttgttttcc

catcagctatgagttgtagtatttcagactcacgatttgtaagttgattatcagaggaat

atcttgaagatattttttcacaatccatatgtacactatcaaaataagtgtaaccattac

taatcgaatcaatagcacgatttagtttcgccggatgactggttttccatacgcatcctt

response regulatory protein ygeK

tcgcaccggcttcaagcaattttattgaatcaatatacggcatttttgctacaaatgtaa

taatatgagcatctattttatgcgatttaacccattttatgagttcggaccccatcattc

cacctgcaaattctcctaaaataaatatttgggctgcagatttgttacactccgcgatag

ctttcttcagatctttatagcccccaacaacctcatataaatcgggataatgtccgagaa

atccaattatcccatcaatcataaacggctgctgatctgaaactacaattttaatctttc

ccatatattcatcctgagtgtaaatgcaataacgcattcatttataatcatcccttccat

tattattataagcaaaatccaaagaatacattgatgaaataataatgaaatataattaaa

aataaaatttttgcgtaagcagaataacacccgtgttgaaaaatcattaggcgtttaaaa

tattagaattagattatattcaagttattaaaccggagacagtgatctataagtcctctg

#3F CGAGTTATAATTGACAACAT

#3R CAGAGGACTTATAGATCACT

**#4 ~5150bp**

ccatatattcatcctgagtgtaaatgcaataacgcattcatttataatcatcccttccat

tattattataagcaaaatccaaagaatacattgatgaaataataatgaaatataattaaa

aataaaatttttgcgtaagcagaataacacccgtgttgaaaaatcattaggcgtttaaaa

tattagaattagattatattcaagttattaaaccggagacagtgatctataagtcctctg

gattcgtcaaatcagaattattcatagccaaagaatctatttcacacattctttcaacta

conserved hypothetical ygeN protein invasion protein OrgB

aataatgtctggcatgttcacagatttcattaaccttgtcaggggaaaaacagtgattgc

taattaaatattgctcacaattatccacaaattcctgcggactaaattcagcaataaatt

gatctgtaaaaaaaataaaacgtttgtcatcatgatatgaaatatggacattccatattg

acttgtcgacaaaatatgactctacttcaatagacttatctttgaaactggtaggtatat

agagatttaaagggcctgatattgacggtaattttgttacccaactttcaaaaattttga

taaaaacttcaacttcctctgaagattttaacaatatgctcttcagatcgtttatcatgt

ttattctgtttttttctaattctgagctcagcaaacttatcaagggtataaaactatcca

conserved hypothetical ygeN type III secretion apparatus protein OrgA/MxiK

tcacatcaataatgcctttcgcatagccatcataataaccttgaataactttttcttctc

tcttttcctcaatactctgaatttttgcggtggtttcaagaaggatctgttcttttagag

aaactatttctttcaatccatcagaaattattcttttgatcacaacaccatttgcaggag

agtttatcaagctcatctctattttttttcgcataaaatgtaattctctccaaaagagta

gacgcaagtggtaacggcaattgtatgtggtcaacaaaatcaggaaataataaattaaat

cgttgctgcattgccaacggttgttgccttacatagggtaacaacgttgaaaaaccacat

gtgatgatattctgatagttaacgataccaggtttatatcgtgccttttcatttatttcc

accggtatcgctgataaataattccttagcacttcaggaatcttgtaaaaaaatccgcgt

type III secretion apparatus protein OrgA/MxiK

tctgcaaacctttctcgataaaagtgacaaccactgagtaaacagatcaatggaaataat

ttccaattgtttatgtagtaaataagtgaactattcagactgaaatgctcaactgaaaga

ttgtactgtaggagtatcatttcattggtaatagatctaagtaccggattgctaatacgt

gcattattcaaacttactcgttgaggatgaatataactaattggatcatatataatcttt

cttagggcaagattcataacgttctcatatgtctacttattttttaacctgattaaatta

gttagaatagccctgtacttccataacagccagcaagtcgcaatggacattaatgcaagt

aagatagaaaccggcatagtcttatcataagcaaaaataggttcgttaatttcatatgtt

ggtgcttgttcaataatatctcttcgttttgacaatacgactgaaatattctcatattgt

acgcttgcaaagctattaacaataaatctcttaatgtcatttatttttatttctggattg

atatctttttcatatacagcaagtacagaaatatgaattggtaaagcagttttgccacta

tcacctgtatcaacatcgtaactaacatgtactcttgaagaaacaatgccatccataatt

eprK

ttgagagattgctctaaacgctgttcaatagcagaatacaatcttgctttttccgctcgt

ggagaggaaacgagtgcatctgcagggaacatctgcgatatttgaatatcaggcttaccc

gggagattgtagatttttagccaatccaccgcagaagcaaaatccgttggttctacatag

attgaaaatccagttttgccttgatccttcttctcagcattaatattatgtctttgtaaa

acagcaaggacatcattagcttgctgttgatcaagatggttcagtaattcctgctgcttg

caaccgcacaacagcaggataaacaagaataatgatatatatttcatgagcgagtcaacg

tctcaactgcagcgacaatttttcttgctaatgttcccaccaatgaaacctcgacattat

eprJ

aattaagcacatgatcctgcaagactaataatttttcaggagttaacccctttttatcag

accttaatgcttgtaacgcattttgctgccaattttcagtttgtaccgcatagttggcaa

aagcggagatcattttatcatccgggttatcaagattgagaacccggtcatttaaatcaa

taactttcgcttgctgtatttcacgagcagctgattgctcagctctatcaataggtggca

tattgcttactgacataataaaatccttatccagtttagcggaagttttgtatgattgcc

gaatcaagatctttatacgcttttacaacgttagattgcgcatttcgatataatgtatac

eprI

tcagctaatgcactctgatattcggcaaggaatttcgggtcggaaggattggttgctaaa

tcctccaacgccttttcaacttgttggttcagatcatcaacgccctgatcaaattgtttg

ctgatatccataatataaccattccaatctgccataagtcctcctcaattattcctgaaa

tgtccacttgctaccttgagacttctcgaatcggttatctccgtagctaaatgaactatt

atttgtatcctgatttttcaaatttacattaaatcttacatattgtttaccccattgttt

ataatattcgtcgacatagttattaattttatttaattcactgtcgcttaacgtaccaat

gatattgaaatatacattgttagctgttttatatttttcataagaaagacctattgccga

caatgtttcaattgcctgactttccaatacattatcatctaataccgcaatacttatatt

eprH

gctggcatatggcatagtttgcagtaatcctttaataagattatcgattagttgctgtgt

gatagcattccgttgcttactcactgtcagtcttggattactggcatctaatagctgtag

tttaaaatattgtagatctgggaaattatcagcaagccatgaaaaaattcgtatcgattc

atcatcaggatttatcacccgtactggctttcctggttgatttttttccagtgcttgttt

gacccagacagcttcatccaaactattggtattgatatagatgacatgatccctaccata

taaaatttcataatttctatttttgtttacaagaattttatcgatatgtctcagctgcgg

atcattactggcattaacaatataaaaagctataaaaatgatagcaaaaagaaaaaatag

cgttacatatttaaacagcgtacttctaaagaaatgttgtcttgttgaagtaatgttatt

ttcaacaatacctggtattgaatactgccaaacttcattttttacttttacagcaaattt

gaggcctaaaacttctatttgctgattcaatgttaacgttctatcttcaggttcttgtgc

tgttaattctctgattgttacaaccagatcagggtcggtagtgattattacctcaaaact

gcctgtaagctctccataaggaatgactatcgtattctctggtaactcagaaaaagaatc

acttcttcctaacgaaacatcattaccaacgataacgagtacacgcccattaagtatttc

atactcgcatccattcaatggtccgctcaataaacgaatggcataagattccaataagtc

ttgtgataagaatttatcattattttccatgaataccacccgctaattccataagctcta

gtttagagtagtgtaacaagacggaaatttcatgaccaaattatgatgacacacaataaa

aagattataattttcgagaaccattttaaatgcctacatacataactttatttgcaattt

aaggtatacaccctattccctcattgatatgcaaatataacaattaccattattttaaat

tttacacgtaatatttaccctaaatgattttagagtgcaggttggcatatgcaagtcttt

tccagtgatgtctacttcactgtaggcactaatgcattattagcatcccaaaaggaatat

putative type III secretion system regulator

tacagcgatttggttgcgttggttgatcttggacattcgttcgttgttattgatgagcac

caacatcgaaatctcaaaccaaatacagaacctgtaaacacccttttgagcaacaatttc

atcaggatcaataagaatattacattatctgatttaacgcatttcctggtttcgaatctt

catacacaaaatgtgtattccactcaagaaccacttacccatgatgagatagatatcctg

cggttatgtgtttcttattcgttaaaacaaatagccattatcaaaggaattgattataaa

acagtatcatatcacaaaataagagccctaaataaacttaacataaaaggtactgttgag

ttgtttatagcactctgtgaatgggataaacattattttaaactccaatcttgcataaga

gaaagttgatatgtcattagtaagatataatagtaaaatcataataactgtagcgctgat

atcgttgctctctgcatgcaccagagaaaattcattaaaggatactcagaataaaagaag

tggcgaatctcaagcgatagtagatgcaagacagttctttagcgaacacccggaatatat

atcatcagcagaactagagcagaagctatatcgcgaatttgcgcaggttactactgttcc

agcttataaagagatgagtatgtatcaattgcttgtcatttcgcaagatcgtttaacaca

ttaaataagaatctacacgctttttgcgtgtagattcttataaatttccgactatgatag

atgtttaatttttttaattattgtctttattctcgctttttgtgtcttcaccttctttga

#4F CCATATATTCATCCTGAGTG

#4R AAGAATCTACACGCAAAAAG

**#5 ~3500bp**

atcattactggcattaacaatataaaaagctataaaaatgatagcaaaaagaaaaaatag

cgttacatatttaaacagcgtacttctaaagaaatgttgtcttgttgaagtaatgttatt

ttcaacaatacctggtattgaatactgccaaacttcattttttacttttacagcaaattt

gaggcctaaaacttctatttgctgattcaatgttaacgttctatcttcaggttcttgtgc

tgttaattctctgattgttacaaccagatcagggtcggtagtgattattacctcaaaact

gcctgtaagctctccataaggaatgactatcgtattctctggtaactcagaaaaagaatc

acttcttcctaacgaaacatcattaccaacgataacgagtacacgcccattaagtatttc

atactcgcatccattcaatggtccgctcaataaacgaatggcataagattccaataagtc

ttgtgataagaatttatcattattttccatgaataccacccgctaattccataagctcta

gtttagagtagtgtaacaagacggaaatttcatgaccaaattatgatgacacacaataaa

aagattataattttcgagaaccattttaaatgcctacatacataactttatttgcaattt

aaggtatacaccctattccctcattgatatgcaaatataacaattaccattattttaaat

tttacacgtaatatttaccctaaatgattttagagtgcaggttggcatatgcaagtcttt

tccagtgatgtctacttcactgtaggcactaatgcattattagcatcccaaaaggaatat

putative type III secretion system regulator

tacagcgatttggttgcgttggttgatcttggacattcgttcgttgttattgatgagcac

caacatcgaaatctcaaaccaaatacagaacctgtaaacacccttttgagcaacaatttc

atcaggatcaataagaatattacattatctgatttaacgcatttcctggtttcgaatctt

catacacaaaatgtgtattccactcaagaaccacttacccatgatgagatagatatcctg

cggttatgtgtttcttattcgttaaaacaaatagccattatcaaaggaattgattataaa

acagtatcatatcacaaaataagagccctaaataaacttaacataaaaggtactgttgag

ttgtttatagcactctgtgaatgggataaacattattttaaactccaatcttgcataaga

gaaagttgatatgtcattagtaagatataatagtaaaatcataataactgtagcgctgat

atcgttgctctctgcatgcaccagagaaaattcattaaaggatactcagaataaaagaag

tggcgaatctcaagcgatagtagatgcaagacagttctttagcgaacacccggaatatat

atcatcagcagaactagagcagaagctatatcgcgaatttgcgcaggttactactgttcc

agcttataaagagatgagtatgtatcaattgcttgtcatttcgcaagatcgtttaacaca

ttaaataagaatctacacgctttttgcgtgtagattcttataaatttccgactatgatag

atgtttaatttttttaattattgtctttattctcgctttttgtgtcttcaccttctttga

atttatcttctgaggggagcagttcatctggaacaggttgtccagcattctccacatctt

caagccacagcagaagacgcaagatctcatctatattttcgaagctaacataatcgtagc

**SpaS**

YscU/HrpY family protein type III secretion protein

gacgatgggtagcataaatttttcgtgctaattttttatctgtaataattggtattccga

tttcctttgcatatttccttacggctaatgctacctcattagtttctctcacagaaatca

atggtataggtgaaagatgtggcttaaagtatatccctatggcaatgtgcgtagggttgg

caatcatcagacggctattactgacatcagatttcaattgctctgacagaatttcttgat

ggcgttctctgcgtttagacttaatttctggatttccttcttgttccttgtattctcttt

taacttcttgtttatccattttcatatctttcataaataaaaaatactcagcgataaaat

caaatattagaacaattatcatactgccgagacaatatagtatgagaaggaacagcatct

ctccccaatctgataataaagaaatgatatctccatcaagagttttaaaaagcagtgatt

tatggttactccaaaataatttgatcgccaatgcaaaaaaataatataaagtattgcttt

aacaaattcttttacggtttttaagccaaatatacgttttaagccatttactggatttaa

tgaatcaaacttaaacttaatagcttcagttgctaattgcattttactttgaaaccatga

aacaagtagtgttgtaaacatacaaaccagcaaaaaaggaatgatcgttttcaaccaacc

tatccccatagctttaaaatagtcccaaatatccggtttagcgttatgatcaaggatgta

ttcaagaatcgacataatgtgatgcacatcaaatacatatccaagatataaagtacccac

aagcattattacagagactgttaagtctctactttttagaatttgaccttttttagaagc

atcctggagtttcttttgtgttggcttttcagttttatttgccatgaaacactctattta

tcaatgtataattgcaattgttctgggaaaaaggataacggcataactcgttcagccagt

attgggccaaaataaataattaatattaaaaatgccagcgcacttttgacagtcagtgag

atggcaaaagcatttagttgtgaagcataacgtgccagcaatcccaggacagcttcaccg

cccagcataaccgcaatcacggggcttgcgtaaacgatagtatgagtcattatattatta

atatatgagaataaaggctctaatttaggaaaattaaaatttcctgaaggccataagtta

taactttgccatagagtctccagaataaagttcaatccaccgttagtaagataaactgct

gcagagaaaaggttaaataatcttgctaattccgatgtatcgacaccagtagccggatcc

aaagtactacttaacgttgcaccacgttggttatcgataaagctgcctatagcaaggaaa

atccaaaatggggatgctaataagcaagcaataaacaaccctatgataatttctctggca

gcaaacaaagcgatatctatttcatttagggtagataattcatagtggtaatgctgaact

aatgcacatgataccactattattattggatgacggactactgttggtatattcccactg

gcgaaaaatggtaacaaatagaaagttggtgccaatcggcaaaaaccgagcgctgtagca

gccagcagactatgcaactgatagagcatcgcctcccccatgctatcctctcgcgaatgc

#5F ATCATTACTGGCATTAACAA

#5R GCATTCGCGAGAGGATAGCA

**#6 ~4000bp**

atcctggagtttcttttgtgttggcttttcagttttatttgccatgaaacactctattta

tcaatgtataattgcaattgttctgggaaaaaggataacggcataactcgttcagccagt

attgggccaaaataaataattaatattaaaaatgccagcgcacttttgacagtcagtgag

atggcaaaagcatttagttgtgaagcataacgtgccagcaatcccaggacagcttcaccg

cccagcataaccgcaatcacggggcttgcgtaaacgatagtatgagtcattatattatta

atatatgagaataaaggctctaatttaggaaaattaaaatttcctgaaggccataagtta

taactttgccatagagtctccagaataaagttcaatccaccgttagtaagataaactgct

gcagagaaaaggttaaataatcttgctaattccgatgtatcgacaccagtagccggatcc

aaagtactacttaacgttgcaccacgttggttatcgataaagctgcctatagcaaggaaa

atccaaaatggggatgctaataagcaagcaataaacaaccctatgataatttctctggca

gcaaacaaagcgatatctatttcatttagggtagataattcatagtggtaatgctgaact

aatgcacatgataccactattattattggatgacggactactgttggtatattcccactg

gcgaaaaatggtaacaaatagaaagttggtgccaatcggcaaaaaccgagcgctgtagca

gccagcagactatgcaactgatagagcatcgcctcccccatgctatcctctcgcgaatgc

taaattcagcatttcgataccaaacgaatataatttttcaccataccatccgctcattaa

**EpaQ**

type III secretion apparatus protein

needle complex export protein [Yersinia pestis CO92

aaaaaagcaaatacttacgcacaataatttgacgccaaatggcaacgtctgttcttgcaa

ctgtgttaccgtttgaaaaagccctaccaataaaccaacaaatgtcgcaactgcaatagg

tcctgcagacataactaaaatcagatacagcgccctattccccgcaaaaacaatgtcgtc

catattttttcctcagggattaatggataggtcaaaatattgtaaaatgagtcctttcga

taacattgtccaaccgtccatcgcgacaaacagaattaattttatcggtgtagatatggt

**SpaS**

type III secretion protein EpaP [Shigella sp]

type III secretion apparatus protein, YscR/HrcR family

taccggactcatcatcatacctaacgttaacagaacgctggatataaccagatcaactac

aacaaagggtaaataaatataaaaacctataataaatgcactttttatctcactcaacgc

atatgccgggagtaacgagaaaattgaaatattatcatcatcgattatctcttcattgtc

ttcagaactattcaccttctgaatcttttcaaaaaagctaaccaactccggttcagaata

ttttatcaaatatgatttataaccactcatgcctgtctcaacaaaattaacaaccgacgc

aacattattgaatgaaagattctcattctgactgttattatatatttccttcccaaccgg

catcattacgaacattgacaatagcaaagctaccccattaagtgtcatatttgatggcac

ttgctgaagtcccaaagcgtttcgtacaataacaaaaacaatcgagaatttaataaaaca

tgtcccggatgctattataaacggtaatagtgtaaataatgataatatcgctatcagcga

aatgctatttgacattattattccctgataaccaagaatgaatttctacgcccaatttat

catccacttcaacaagttcgccatagccagttagagcgccatttacacgtatttctatat

ttttctccgcttcaggaagcaaagatataattctttttgcacagagatcatctatttcat

atagattcattattttctttccaagaacaaactcaatttttacaggcaagtcttcaaaac

tattaatctgctgatagtcattttcatcattaatctcgatttcgctttttttgatatcaa

**SpaO**

type III secretion apparatus protein, YscQ/HrcQ family

agtcatctgtttcaaaatcttcctcgtactcaaaatgatcaaccatttttaactcctctg

gataaattaaatcacaaatctttgtattataaataaccgcataagcaaaattatttgaaa

ttaataaaacatcaccaacttcaatatcaacaatggttctataacatgttttactaaatc

caattacaaaatgaatgggccaatataatgattttttgtctagcgttggagcgttttcgt

attggcagactgtgactttatctaaccaaatatttaattcccctgtccatattcttatca

gtggttgagctggactcaatactccatcaaatactttaccgacacacaaacgattatatt

ttagttcaggatactttagatttaacgggatactagataaactgagccattcccctatta

acctttccgtttctggcattaaagccatcccagcgagctcggcagaaattaagggaaata

actctcgaagtaacacccatccagaccagctgccgctctcttctgctatcggtagccatt

tggcgctaaattcgggcattaataacgcaacatcttcgcccttttctttccaattttgaa

aagtggtttcaaaggaatgagtgtttccatttacttttctcagaccgaacattaactttc

gtccttctgatcgttttcgaatttaccttcattttcttgctcatcatgaggctgatgtcg

ttgcccttgtcgttcatcttgctcagtcaatacccagcgttgtgctgtaacatcattgtg

ttgggcttcatggagtctgtcggagacaagtgtgtctgatggttttagtcgtatcccttc

**EivJ**

Type III secretion apparatus protein

actactttccagaatccggactgtatgattttgtccccaacgctgaaactgatatgtcaa

ttcattttcgcgcgcattggcattattagcagaattatttatatcattctgattgagcgt

atgaacattcctttgcttttcgatatgttgagtaaacatggttctgttatcagcaaacct

tctttcgttagtaatgtctaatgaatgggcctgcggtccatttattttgtctccatcgcg

gtattttttttcacgatctttattattactgtgtttttgatctgtgatgtattctgtatg

gataccattatggattttttccttaaattgtatcgttttattagatggttcagaatcatt

cccctgatctcgtgaagtcatattgagaattttctttgtagaaaatttctctgttccatt

tgtttgcaaagaaagagatggattcctgtcaacctggagagatgatttgttgtcaataga

ctccatgtgcccattttttttccccaacgtagaaaattggctatcctgagtctgacgtaa

**Orf**

Type III secretion apparatus protein

taactggagaatggttccagcgacaatctcactcatttctttattttttttgtttttctt

taagaaactgtctaaatctgaagcagaattatcgggtgtgtctgtgtttaaaattttaca

gtgttcaattttcttgacttcgtccattatatttctcctcctgttcaagctcgtcttgat

aaagcatctgatttaaaagttgttttttgatgcgcttctttagtcgatcgtacttttctc

cttttagccaccagtgttttctttgtgcttgctgttgaactatttccttgttaagtattt

**EivI**

type III secretion apparatus protein

ttcgccgctctgctattttttgcttttcaagatttatctcagcgatctgctgctgaatga

ctgcaactttacgtaacgagtaaaatatatcctgtcgaaatacaacttcgccgagtaact

ctttcacatctaacagtgactgtaggtttttaagttgcatatcatacaggcgttcctttt

cggctaacttttctttttctgcatttaacttttgtaatgatgcttcacatgcagccaggc

tctgcgctgttcttctgataagtcgattaactttacttaacaattcgttcaagctctttt

#6F ATCCTGGAGTTTCTTTTGTG

#6R AAAAGAGCTTGAACGAATTG

**#7 ~7750bp**

ctttcacatctaacagtgactgtaggtttttaagttgcatatcatacaggcgttcctttt

cggctaacttttctttttctgcatttaacttttgtaatgatgcttcacatgcagccaggc

tctgcgctgttcttctgataagtcgattaactttacttaacaattcgttcaagctctttt

agcgtttcactcattggcattttttcattaactgactgtttcaaccaatttgtgagttta

ggccgtgcattcatagcaaaatcattttcagcattttgcccagcgcgatattcccctaaa

tcaataaacacctgtagatcttccaaagttgtgaggatcttccttacccgcgcagcatta

tctctgtgtttttcatcagtgacctgaccaaatactcgactcacactttttagtacatct

attgccggataatgaccttgcccagcgagtttgcgacttagatagatatggccatcaagg

attgagcgaatttcatcacccagtgggtctgattcatcctccccctccagtaatacggta

**SpaL**

ATP synthase SpaL

taaaacgcagtaatacttccttttagtgttggtcccggcctctccagtaagcgaggtaaa

ctgtcaaaaacagaagcaggataaccacgtctggcaggtggttcacccgctgccagcttc

atatcgcgtagtgctcgtgcatatcgcgtcattgagtcgataaaaagtacaactctcttt

cctcgatctctaaaatactctgccacggttgtcgccatcagggcggcattgcaccgatct

actgacgaaaaatcagaggtggcataaaccaaaacacattttgctgcatttactgatttt

ttcagcgattccgcgcattccgtaacttccctaccacgttcgccaatcaagccaataaca

aacacatcagcttcagtgttgtttacaagcatatgcattaaaaccgttttgccacagcca

gccgatgcgaaaatcccaacacgttgaccaatgccacatgtcagtacgccatcaatgact

cttatccgtgtctctagtggtgtattaataactcgacgttcctgatacgacggtggcagt

tcatcaatcaggcgtaaatttcccctttcgcctggagtgtccgggacaaacctttccata

atttgaccagaagcattcaaaactgaaccaagaaaggcatcactgatctgtatggttaac

tgctctcccgttggtttcaaaactgcagtacgtgtaagccccacagagctgcctataagg

tttaataatgtctttccattgcgaaagccaacgacctgagccctggctatacgttcattt

gcttgccagctcgcataaatttcgcatacttcgcccactgaaacgtcgtcaagctctgct

tcaatcagcgagccgtttatgctatgtaagtagctgtatttatttaatagttttaatttt

ttcataccagcatgctccatatcgcaagacattatcctaaatagttttcagaatattaac

gggaacattttccgttagttccccgtacgaaagtacttcgaggtctcgataactagattc

aataagttttttgataaaacgacgaatatccacagatcccagtaaaataatgtctttaat

aggaatattgattccagaaagtgccagtttaaaattatccagaatcatctcagcttcgga

tgcatcgagattaagaaaagtaccgccagcggtaggcctgacaccatctctgattctgtc

ctctatttcaggcgaaatcacaattgctttaatttctccagaatatgaaaacttatggca

**EivA**

invasion protein InvA

type III secretion system protein BsaQ

HrcV family protein type III secretion protein

type III secretion protein SctV

aatgtagcgacctaatgcacctcgtacatgctcgacgagtgtaattatatctttctcccg

tggactccataatgctaaagcctccataactaaacgcatattgcgcactgaaattcgctc

ctggatcagacgctgaattacctctgaaatgcgctgcacggttatatatcttaatatttc

ttttaatagatcaggatacttcatctctaattgatcaagaatatatttggtttcttgtat

accaaaatactcattaatattatgcgccagacatacagataaatgattgtacatttcatc

catggccgtgcgagtataacagccaatttttgttaatttttgggcatcactttttgtaac

ccaataatattgttcaccattactatttacgatgacaggtttcttgatcaaagtcgaaac

cacttcatctgaatattcaaccaacaagacgagatcatgataaattttaaattgcgaggc

tctgacttcatttagcattaatatgacatcttcatcattaaggccttccccctcgcgaat

gacaataccgggaatacggataccatactcaataaaaaactgactacgaatcctatcagc

aagattgatttcagtgtattttttagcctgtacactattcataatcaaaaccaagggtac

cgtttcggtaataacctgatcaagtttattaataatacctaatgaaatatcatcatcctg

attatagggttccaacccaacggtgatatcgccctcaacaaccgtttcttcaaccccttt

ctttttgaatttaaagtaaaaataaattcctaagattactgccaaacataaaaacacaag

aaggggaaaacctggaagcaacccaatacttagcgcgaggacacatgtcacaataatgac

aaaagaattacttaataactgggacattatattttgccccatattatcactatcgccatt

cacgcgcgttacgataaaccctgcactaatagcaattaataaagctggaatttgtgaaac

aagaccatcaccgacggtaagaattgtgtagacggtcaacgctgtcgagaaatccattcc

attttggcctactcctactgatagcccaccgataatattaacaaagataataataatgtt

tgcaatagcatcacctttgataaatttcatcgcaccatcaaaggaaccatacagctgact

ctctcgttctaatacgctacgacgttcttttgctaaatcggcatcaatgatcccggcgcg

tagatcggcgtcaatactcatttgtttacctggcataccgtcaagcgaaaagcgggcagc

aacttcagccacacgttccgaacctttagtaatcacgataaactgaacaatagtaacaat

ggagaaaataacaaaaccaactaccaggctatctccaataacgaattcaccaaaactcgt

tataatttctccggcgtccgcctcaagaagtataagtcggctggtactgatcgccagcgc

caaacgaaataacgttgtgatgagaagaattgatgggaatgaagaaaaacttaagatcct

atcaacatagaatgatcctaaaaacacaagtattgccaatgttaagttcagcccaatcaa

aaaatcgattaagtaggtgggcaatggaattatgagcatggcgataatcatcaccattaa

tccaagaattattaattctggatgactccttaaccctatcaaaactttgttaaacattga

acattaccttcttccatggttttttattttgttttttattactgttttgtgtatcaaaga

gctctgctgcaaatgttacttttattgcctgtttacaacactccctaatctctaataatt

gttcattattaataaaaagtgaaaatggaagatttgtacatccgctgtaaatcgccttca

ggagtaacgttttgtctttataattagcatctgcgggtagggttaccagtgcatttttca

ggagttcctctgcagtctcaggaaattttattaacgatatcagaaaaaatatccaataat

cttctgcgctaataaattttttaattattgggttattcattaaagttttcagaaataaag

**EivE**

type III secretion regulator YopN/LcrE/InvE/MxiC

cctctgccgactgaagagacaatagttgtgacaacttattgagaaacataccaaattcca

atgatgaacagctcgcatcaagtgcatttatatctgagaaaagcgtctctttgataaatt

ttgtcaccagcattctattttgatagccataactaccaatccattcgacataagtatcta

cagcatcgtcttctgccattaaaaattgtcgataacttgcccttagtaattttggattta

atgacaacgcctggccaaataactttgcttttaatgccgaattaataccgctattaatga

attttttagtagtctcttgattaacttttttaagtaatgcttcagcatttagccgcacat

tttcagatatttgttttttacgtaaaattgcacgtaatacaaccaccagatcgctttcat

ctggaaataattttttagcaaagctatacacttcatagtcatgggtagttgctgccttaa

gcgcctccatgactttatcggttttttctattggctgatcttccaggacatattcttctc

cgtccagaaagttggccagttgttcttcgtagatttttttcttcataaactgcgttaacg

ctgctgacatctcgtcagtagcttgtaagaatcgagcttctttaccggcgggatcatctc

ccattttaggcatttcagcttcaatatcttcattatcttctgtgataatgtcctctaagc

gagaaacttctctggctctttctaatacaccgacatgttctacatgaatcgccatttcac

tatcctccgacgacttcacgattcaggtaagtttttatccatttctgcagtaattcatcg

ttgatttctttattttttgccatttgctgtgttattgctctggcgtcaaccgcagcctca

ttcgcattattcatcatgcgctcatcgatctctcttggctcaataagaaagacacgaacg

atgttattcgcatttgttccttcatatccaaataatttaccaataaatggaatactgcct

aatattgggatcttgcgactttcataggtattcgtatctcgtgtatatccaccaatcaaa

agacttttcccttgaggcacgcgcgcaatagtacttataagcgttctaccaacctgaggc

agatcatcaacattggtcttgtcagaattgatttcattgccatcttcaatattcaagagt

aattcaatttgatttcgcgaagcaaatcgtggtagcacgctaatcatcgtaccataggtt

**EivG**

protein invG

YscC/HrcC family type III secretion outer membrane pore

acttcatctaactccgcagtacgctcgccgactaatttagtatagaaagtacggttatta

tcaaatatagcaggaatattctcctgtgttaaaacaacaggacgagcaacaacggcggct

cttcttttttgcgcaagtgcctgtattgttgcaataaactgtgtcccatccagggtacta

attgaacctgaattattaaatgatgcacttaagctggatccaatttttatcgtgcccgac

cagtcagctcctaactgttctagatcagtcttatcaatatctattatccataaagataat

tcaatgtgacgctttggaatatcgagtgtagctactaacttctcgataaaatcaacctga

gatactgttcctttaacaagtaaactgttggtatcaggatatgcaataatttgtatatcg

tcagtctctggcgctccagctgtcgatattattttctctacttgtagatcatcatcttct

tcagcaccaggaaaaggaggcatacgtgatacgttttttagttgttgacgacttgaaatt

tcagaaagtacgttaaccccagtgctttgcttaatgttattatttaacaacgtcgatagt

acttttgccatgccaggaataactatcttttcgccacgtaattcataagtccgatcatta

acgaatgtgttgaccaggtgaataatccctactttattgcgtccgatctcgatgccatca

ctgttttgctccattaattttgcagcattaacgaccaggtcaacatacaccggtggccct

gaaacatagaaagtaccattgccgccccctttaatttcataacgagagttatacagaccc

gatttttttaaaaaattattaaactcattcgtcgatacctttctcagattaatcagcgca

ttacgcatctctgatgagtcatagatataaatagctttgccatcatcgtaccaaataagt

cccatctgcgccgacaggcgttcaatcaaggcatagggttcggttaaatcaaaatttccg

ctgatctgtttcttcatggcaagcttacttactattgtaggcttacccgcatatgatgaa

accgtttcaaaaaaactacgtagattttctttatttgctacgtatccattcgctgttgcg

gcatatgcaccaggagtcaataatccattaaaaatgcataaggctgaaattaatataata

gtaatacgtaattttattttcataactttttacatagttctctcggtgtttttcctaacc

ggctttttacttcggctgaaaagtgtgatgaggatgagtagccatatttatgcgcaatag

ttgtcatatcactgtttccttcaatgatttccaacacggctctggcaactctccagccac

**EivF**

putative type III secretion apparatus regulatory protein

ataattccgttttaactttaccacccaatgcataactacataaacggcgaaaatgtgaat

acgatacgccgtaaagctcaccaagatcatagagcgaagtctgtatgagcgattgcgata

ataaatagcgaaccagtttatagcattctgtttttcttagatgctgaaacagaatttcat

taatattatcgctgctcagataagtggctaaaaaccaggcttcacttgcaactttactga

atagcacaacttctgatgcaaagggcaaggcatcagcaaacaactcatggcgttcccttt

ccacactccccgttgactcatcaataaaagctaataatttactcaggatatcaatgcgaa

gtggatgataaattactttccctgcagttacctcaatttggcttcggtcaatgagcagta

atatgtcttccactaccattaatttttgatggttattaccaatcgtaataacaagggtta

catccttgttttcatttccccgtaagaaccacatttcagggtccagtagggttaacttct

gtccttgaagcaaaacattcattggaagtaacagcccttcttcaatcatgtttcatctcc

ttattccatattctcattctccataaacttgcaataaaaacatgattaaaaagtgatgat

tcctatttataaaataatgattttgttatgagcgaatagctatcagccacttacagtagt

aagtcattctgtccatatacagaattgcaactgttgatatgttttacttaaacatacccc

gtatttcttataacacttgtttggtgactattttcaataaaaatcatatttgttagttac

acattaacgtactgcgagtgttttgttactccccgcatatattttattctctttttcgat

tctatttttcttaatcgttcatataattttcttgttagtcggatttacgaatcatgagac

**ORF**

conserved hypothetical protein

ttatgatggatggaatgaggctaagcggccaggtcaaccctgtaattactgcagcaaaca

accgatatgttactttttcttccgagaagataaatattaaggtcgatgtcacaaccagac

ctatcaggtaagctaacagtaatttattgatcacttttacctcccttacgatttctcttt

gagcggtccttttaatccaggaccgcctcattatgaaaactatttatttccctcatcatg

#7F CTAACAGTGACTGTAGGTTT

#7R CATGATGAGGGAAATAAATA

**#8 ~2500bp**

gtccttgaagcaaaacattcattggaagtaacagcccttcttcaatcatgtttcatctcc

ttattccatattctcattctccataaacttgcaataaaaacatgattaaaaagtgatgat

tcctatttataaaataatgattttgttatgagcgaatagctatcagccacttacagtagt

aagtcattctgtccatatacagaattgcaactgttgatatgttttacttaaacatacccc

gtatttcttataacacttgtttggtgactattttcaataaaaatcatatttgttagttac

acattaacgtactgcgagtgttttgttactccccgcatatattttattctctttttcgat

tctatttttcttaatcgttcatataattttcttgttagtcggatttacgaatcatgagac

**ORF**

conserved hypothetical protein

ttatgatggatggaatgaggctaagcggccaggtcaaccctgtaattactgcagcaaaca

accgatatgttactttttcttccgagaagataaatattaaggtcgatgtcacaaccagac

ctatcaggtaagctaacagtaatttattgatcacttttacctcccttacgatttctcttt

gagcggtccttttaatccaggaccgcctcattatgaaaactatttatttccctcatcatg

ataaggttggacatttctttcaacataagagtcattgaggctttcgaccagtttagcgga

atgatagggcttggcgcttcatgcaaagttcctgacttgtggatatagttatagctttcg

ggaagtgccatttcaggtacgctcctgccatttgcaccaatcatattctcgcctgttatt

**ORF**

conserved hypothetical protein

tgggccaatgatctattcataaattgcactgagtcattcaaatattcttcttttcttgat

tctttataaacaattgctgctgattttgcataccatgagtcaaaaaaccattctgcttct

gttgatggaatgaagctcttttctcttttcgcatgtgaattttgatcggtatcagtttta

atatcattgaaccaaaaatttgccgactgataattatccttctcgtatctttttatacca

tacggacctgctaattttttaactatttttagaacttgctcttttcttctggtgtttatc

tttgaaagatttgcaggataaataacattaagtaaggccgcatcagcttctctgtaatgt

ttatctttttccagataaccaggggattctccccctaaatcaagttgtagcgttattctt

tcataacctttatctatgaggtggttcaacctcgttgttgaaagaggttcatccagttca

ttggctttagcttcgcgcagtaaatcagagataaaaacagagtctttcttcgagagtaag

ttcgacaaacgttccagtccagaggtcaccagtgcaacggaagatgtgtttaatcgggca

tcttcttcccacgctccagaatcttccataatatagaaattggctttatccagataagca

accaacagcgcaatcgattttaatcggtcacctttttcccagtcttcagcgttaatagta

ccagtgtcgatggcttgtataaggagatcaagataaagacctaaagcatcattttgttta

tggttccacagctgcggcttgccttcttcctgtacatcagccatcacgggggagttgctg

tcaaatcggatatgtacagcattcatttgcccaggaataccatccagtcgttttgggttg ctaatgacgtcctgcatccgcttaatctggtcgggcgtagacatataatcccacagagtg

agcagaaccttttttgcggctacactatttccctgatctgatactaatgccatatagccc

cataaactatccctcagccagacagcatcatagtgggtttctgccgctgttttgtcatct

tcgaaagtagaagcaagaataaatgttccccagggtttctccgtcaacgttaactttcgt

gtattttctgttaatgttagcaatccttgaatttcgtctttggtatagtggctctttatt

**ORF**

hypothetical protein

tcattttttaatttggctgagtgaatcagaagacgataattaggatacttatctggattt

aacgataaataacttttatcagttgctgcccccgtggaattaagaatgttaaatacagat

tcactcgtcacttctgcccctgtcgcctggcatgtgccgcatgaaaatataccagcgagc

agaagagttaatgtagatttccctttcataaaagattccatcaatcgtttgttaaaagcg

tagataaccacttatttaacaacttgaaaataaggtattttacctgggttgttacaaatg

gattgcattgcgtaaacgcttttatttacaaaaaaatggggaagtattacggcgattgcg

aaaagtctgaagcgattgaattaagataatatagaaaatcagatacaacaaaaatggc

**tRNA**

aggttgatgaggtgaagataaatt

#8F GTCCTTGAAGCAAAACATTC

#8R AATTTATCTTCACCTCATCA
