# Supplementary figures and images for: The Escherichia coli Type III Secretion System 2 Has a Global Effect on Cell Surface
Source: mBio. 2018 Jul 3;9(4):e01070-18. doi: 10.1128/mBio.01070-18 (PMC6030553; doi:10.1128/mBio.01070-18)

FIG 2 Appendix

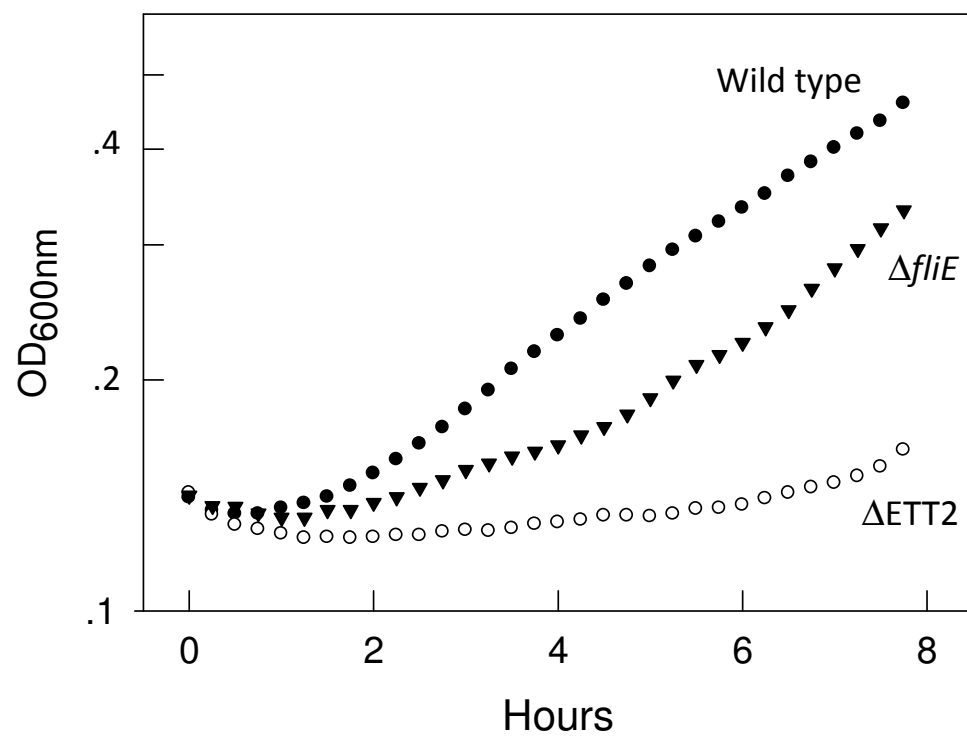

Supplement: FIG S2 [file mbo003183965sf2.pdf]
